# A proposal for realizing cognitive functions using traveling waves, phase-locked and synchronized patterns, holography, neural mixing, and single sideband communications

**DOI:** 10.3389/fncom.2026.1833027

**Published:** 2026-07-21

**Authors:** Janet M. Baker, Peter Cariani

**Affiliations:** 1 Massachusetts Institute of Technology Media Lab, Cambridge, MA, United States; 2 Harvard Medical School, Boston, MA, United States

**Keywords:** brain waves, coherence, correlation, coupling, oscillations, radio communications, spatiotemporal patterns, wave computation

## Abstract

Waves are fundamental. In our view, waves in the brain may constitute and drive organized neural activity patterns on individual neural and population levels. Their interactions follow basic physical principles. Taking a comprehensive, temporal and spatiotemporal perspective, we endeavor to explain multiple brain functions and behaviors with a unified mechanistic approach. Starting with neural architectures, traveling waves, and spikes as the basic signals of the system, our multidisciplinary theory proposes that precise temporal phase-locking codes, spike coincidences, phase relationships, recurrent networks, temporal and spatiotemporal population patterns, pattern correlations, their interactions, synchronizations and couplings play an essential role in determining brain dynamics at multiple processing scales. Analogously to optical holography, it posits that traveling brain waves convey spike timing information, interact to form distinctive time/phase interference patterns and spatially distribute these widely. These in turn can interact with other traveling waves producing yet new spike patterns. Traveling waves also serve to selectively reactivate/refresh existing patterns. Spatially distributed temporal spike patterns and representations derived from these, are used to code, process, synchronize, integrate, and decode objects (e.g., sensorimotor events, concepts, etc.). We apply established physical principles (e.g., wave dynamics, holography) and signal processing principles (e.g., linear additive operations, and nonlinear, multiplicative frequency mixing). This theory proposes that oscillations may serve as signal carriers for communications in transmitting progressively processed signals through an emergent cascade of neuron mixing stages. Such cascades closely correspond to intermediate frequency (IF) processing stages in broadly used radio communications, specifically superheterodyned Single Sideband Suppressed Carrier (SSBSC = SSB) communications technology. This is illustrated with a numerical speech/language hierarchy oscillatory cascade model. This model correlates signal processing stages with both canonical oscillation bands and associated cognitive stages. Plausible biophysical mechanisms are proposed to realize these processes. Many neurophysiological observations consistent with these proposed mechanisms are referenced. This proposal is novel in suggesting conceptual integrations and coordinations of multiple disciplines that mechanistically trace informational neural signals from inception to conception. Some suggestions for empirically testing these hypotheses are presented.

## 1 Introduction: neural information flow in the brain

Like many other neuroscientists, our ultimate goal is to understand how the brain works as an information-processing system that guides and orchestrates behavior. We seek to identify and understand how information is encoded, transformed, organized, integrated, stored, retrieved, communicated, and used to realize the many diverse functions of brains: perception, cognition, memory, decision-making, bodily regulation, learning, and motor action.

Complementary to biophysical and dynamical systems models that predict trajectories of neural activity, we seek to connect patterns of neural activity with perceptual, cognitive, memory, and motor control functions. These involve signal-centric neurocomputational hypotheses, theories, and models of brain functions that underlie behavior.

The current literature on oscillations, traveling waves, spatiotemporal patterns, and various neural codes in the brain is voluminous. Useful introductions to these fields and references to specific bodies of relevant research are cited in each of the Topic sections below.

Our previous papers have outlined and detailed how temporal codes and time-domain neural signal processing might support these functions (Cariani and Baker, 2022; Baker and Cariani, 2025; Cariani and Baker, 2025; Cariani and Baker, 2026). They have also introduced issues related to potential applications of holographic principles to neural information storage and processing, to oscillation-based neural coding schemes, to nonlinear mixing of existing oscillatory frequencies to generate new, emergent frequencies for communication, and finally the relation of oscillatory cascades to stages of speech and language processing. This paper advances these themes and suggests how some of these mechanisms might be integrated and unified to support a coherent flow of information processing in the brain.

Understanding waves, their dynamics, interactions, and the capabilities/consequences of these, may be key to understanding how our brain processes and communicates information. The proposed brain theory starts with informational waveforms, generating spikes/spike trains, temporally coding these, creating multiplexed population patterns and oscillations which interact dynamically, generating selective associative networks, and communicating largely through self-organized recurrent anatomical architectures, by which they are enabled/constrained. These architectures are dense, heavily interconnected arrays of delay-lines of varied neuronal components, organized divergently/convergently, and structurally modified through spike timing dependent plasticity (STDP).

This theory describes these operations and shows how they may be physiologically integrated signal-centrically. These operations can help us to understand signal/information coding, associative network construction, neural processing, pattern creation, detection, recognition, and communication flow in support of brain functions and behaviors.

Principles derived from holography are applied to enable some of these operations. Holography provides a concrete, technological example of how signals can be represented by using wave interference patterns in a widely distributed, nonlocal fashion. Holography exemplifies many key functions that appear to be crucial for information processing in brains: nonlocal representations, content addressability, pattern completions, The nonlocal nature of the representations further enables broadcast-based coordinations and selective reception by trained neural assemblies.

Holographic principles define how wave interference patterns are generated and how these can be productively applied (e.g., efficiently capturing/reconstructing 3D object images, improving resolution of optical systems, etc.). The aspects of holography that we regard as especially germane are temporal and phase precision, widely distributed representations, unique pattern creation, selective pattern completion, prediction, and content addressability.

Our working hypothesis is that the physical principles and computations derived from wave dynamics, signal processing, holography, and communications, are useful for understanding cognitive information processes. Neural mechanisms based on these dynamics may plausibly drive these processes. This perspective is supported by empirical evidence compiled from many sources.

Brains are continuously barraged by stimuli of all kinds. Most are necessarily ignored. How do nervous systems process these expeditiously, to successfully navigate through an everchanging landscape of time and space, driven by past experiences, goals, and expectations?

Brains can be regarded as hybrid analog information processing systems in which external stimuli (e.g., sights, sounds, and odors) are converted from continuous, analog waveforms at sensory surfaces to trains of pulsatile neural action potentials (spikes) and graded ionic currents. These are subsequently processed by cell ensembles and interacting networks to perform multiple functions that emergently produce a myriad of perceptions, motor actions, cognitive processes, executive decisions, etc.

The time scales on which these various actions transpire can range from sub-millisecond resolutions for dynamic, transient events to seconds or more for slower steady state signals. There is widespread appreciation of the potential importance of fine spike timing, particularly in sensory systems. If fine timing is critical in central stations, then sufficiently high temporal resolution must be preserved in experiments and analyses. For example, in order to accurately detect and characterize events that occur on a 1 msec timescale, one needs to digitally sample an analog waveform at a minimum of 2 kHz (twice the Nyquist Frequency); for events that occur at 0.5 msec, a sampling rate of at least 4 kHz is required.

The thesis advanced here asserts, that for signal representations, codes, and subsequent neural response characterizations; e.g. phase-locking, spike timing patterns, phase/pattern synchronization, that much higher temporal resolution is required, than is typically assumed or applied in many neuroscience research studies. To adequately characterize relevant signals and neural responses, it is important to employ suitable methodologies plausibly capable of capturing and processing meaningful attributes and events, within a consistent framework.

This is a signal-centric theory, that architecturally is largely self-organized; e.g. STDP, supporting associative cell assemblies, one-shot and reinforcement learning, etc. Iterative interactions of signals can determine system operations; e.g. integrate-and-fire, “fire together, wire together” associative networks. Furthermore, the wave dynamics, on which these interactions are based, could directly explain how temporal and phase coordination produce observed neural functions and processes; e.g. synchrony, phase coherence, coincidence detection, etc. The physical principles inherent in holography could explain how widely distributed, content addressable memory can be created, stored, maintained, and retrieved, noise robustly, across multiple brain regions. Standard signal processing and radio communication principles could explain observed cognition-related oscillation state transitions and inter-areal information transfer. All of these could be supported using a temporal framework and perspective. Combining and integrating temporal and spectral analyses for spatiotemporal representations and processing of neural signals is recommended. By applying basic physical principles to appropriate neural representations, many neural processes that support cognition and other functions, might be more easily interpreted and understood, at both local and systems levels.

### 1.1 Organization of topics: toward a unified brain theory

**Figure 1:**
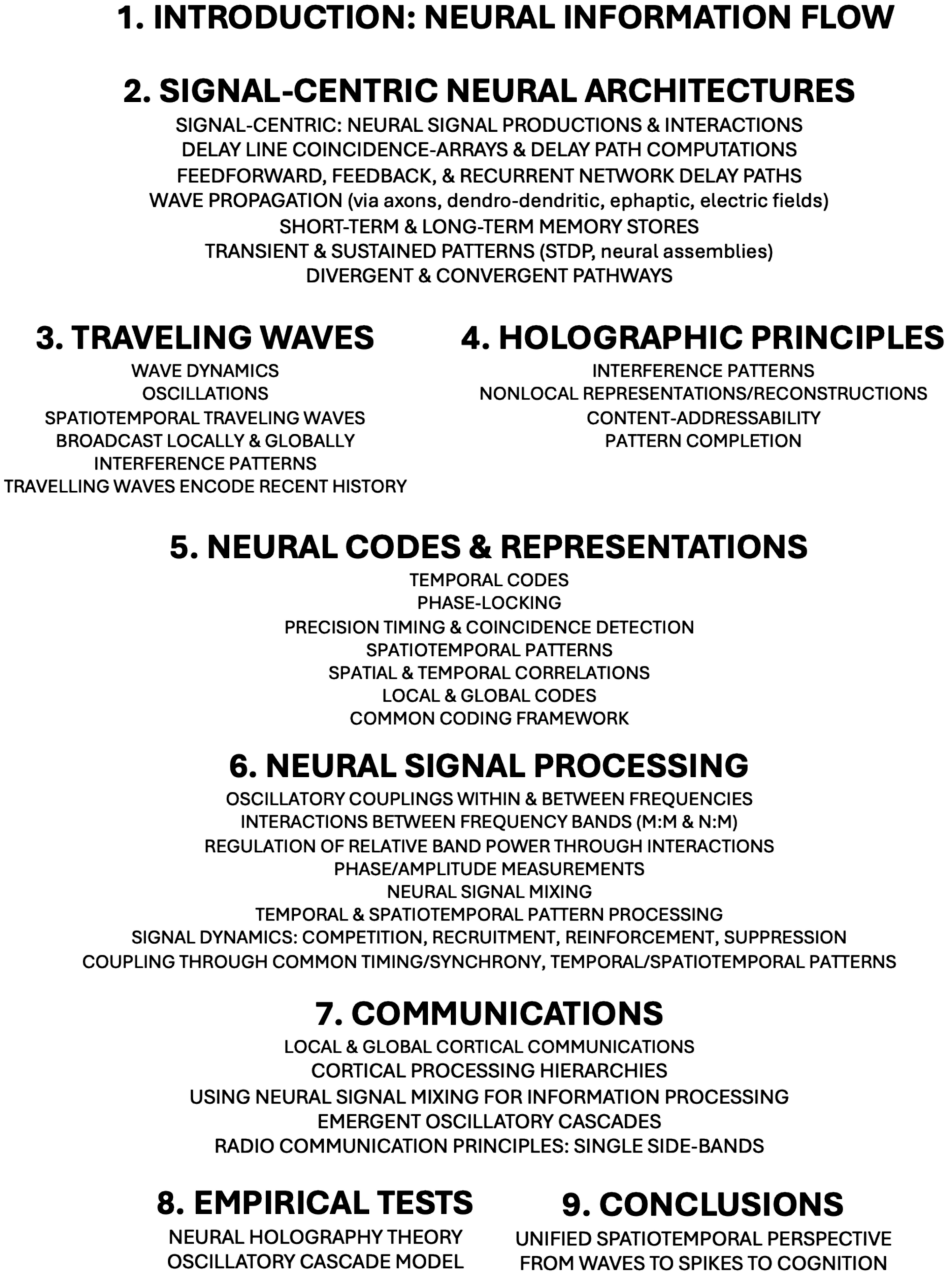
Overview of topics covered.

An overview of this paper is presented in Figure 1. The topics themselves are intrinsically integrated and inherently coevolved for effectively manipulating/managing goal-oriented flows of information with efficient analog processing mechanisms. This paper first discusses signal-centric neural architectures. The neural architecture is largely configured by the analog signals that it processes, and that as a consequence can support diverse information processing operations (Section 6.). It is “signal-centric” in that the information is embedded in the signals themselves and not in which particular sets of neural elements are activated. The neural networks are dense divergent-convergent structures which can be characterized as complexes of massive delay-lines and coincidence-arrays. Their ubiquitous recurrent structures can support memory and other operations. Heterarchical organizations enable the integration of diverse types of information over time and space. Plasticity enables dynamic generation and modification of these structures.

**Figure 2:**
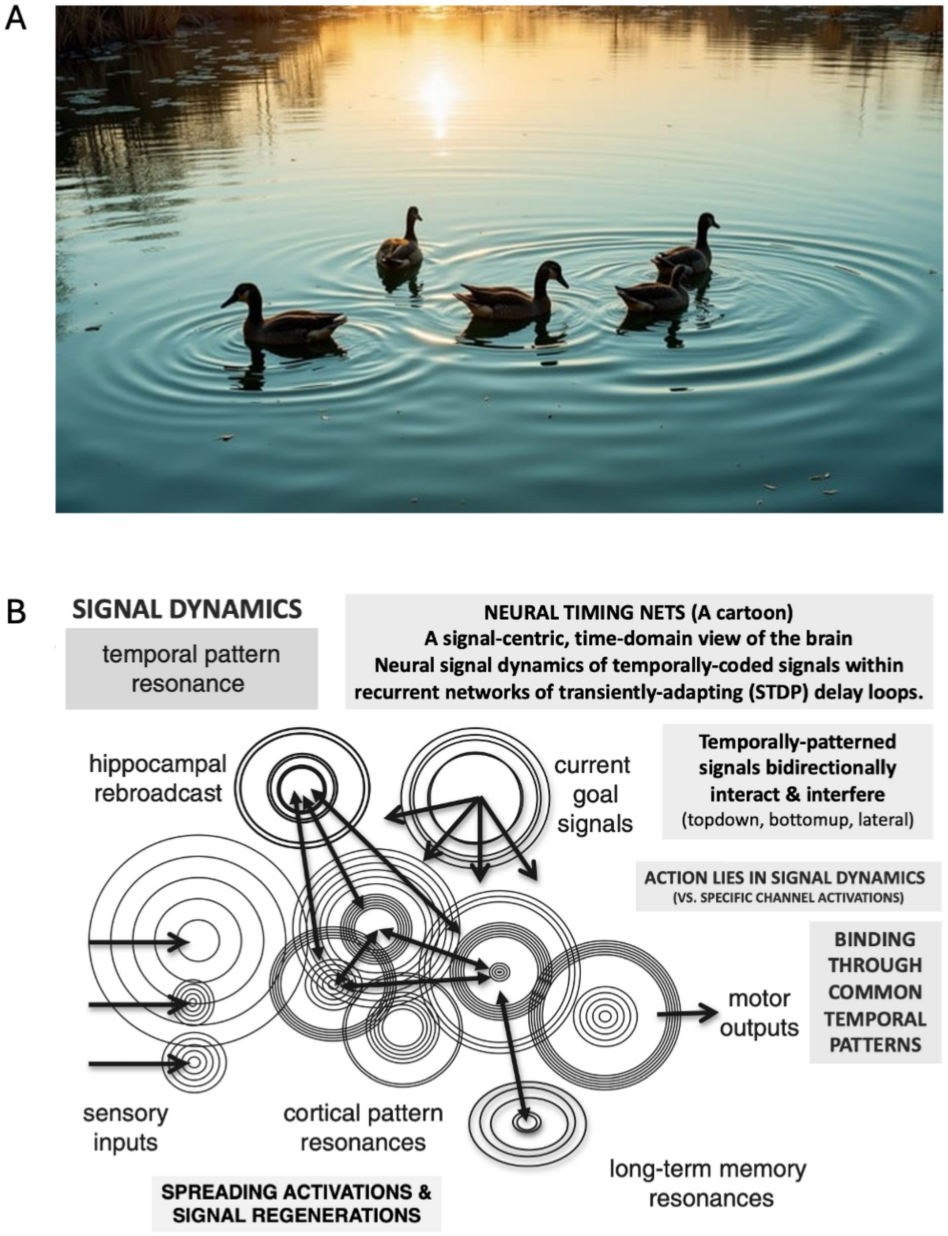
Wave interference patterns and brain dynamics. **(A)** Wave interference patterns in a pond. Photo credit: Tranquil Duck Sunset, StockCake, https://stockcake.com/i/tranquil-duck-sunset_2447582_1468092. **(B)** Neural signal dynamics. High level schematic modified from Cariani (1997).

Traveling waves and oscillations, subject to wave dynamics (Section 3), are fundamental to the propagation, activation/suppression, and selective broadcasting of information, locally and globally. A graphical illustration of these concepts is presented in Figure 2. In conjunction with traveling waves, holographic principles (Section 4) permeate the dynamics of essential neural operations. Interacting coherent waves create interference patterns that can carry and store information in a widely distributed manner. Brains could plausibly use such nonlocal mechanisms for representing, transmitting, storing, and retrieving information. Holographic processes depend on precise temporal/phase interference patterns. Analogously, in the brain these could be carried by temporal codes involving correlation patterns of spikes (Section 5) and phase-coherent interactions (Section 6). Such temporally-coded signals could plausibly be processed by neural architectures that consist of multiplicities of delay paths and coincidence elements (Section 2). These networks include high degrees of divergence and convergence, analogous to interactions of reference and object beams in holography (Section 4).

The spiking-based signals and spatiotemporal patterns of the system can be distributed through population-wide oscillations. Signal pattern interactions serving to associate and dissociate cell assemblies, often quite transiently, is discussed in Section 6. Mechanisms that support local and inter-regional interactions enable selective synchronizations, signal gating, suppression/disinhibition, and matched filter analogs. In the contexts of specific tasks at hand, specific subsets of neural populations can be coupled and thereby grouped by common timing (synchronies, periodicities), oscillatory frequencies, and common temporal patternings at the levels of individual neurons, ensembles, or populations.

Oscillators can be coupled in several different ways. Nonlinear mixing mechanisms similar to Communications Theory principles, such as superheterodyning and Single Sideband (SSB) processing, are discussed in Section 7. In the context of brains, similar multiplicative, neural mixing operations (e.g., multiplicative interactions between two neural oscillations at frequencies F1 and F2) can create new oscillatory periodicities (F2-F1, F1 + F2). These have been experimentally observed. While maintaining lower sidebands, a cascade of oscillatory frequencies of ever lower frequencies can thereby be emergently produced by successive combinations of interacting populations that then can support ever longer windows for informational integration. Such oscillatory cascades and interactions may help explain the various sets of oscillatory frequencies that brains produce under different situations and contexts.

These mixing processes incorporate signal dynamics, reinforcement, integration, the creation of new signals/patterns, and the abolition of old signals/patterns. Additional processing operations include multimodal multiplexing, synchronization among population patterns, grouping and association processes, memory processes, content-addressable retrieval, supervised and unsupervised learning. As a consequence of these processing operations, new high level associative networks emerge.

There is evidence that informational integration and the coordination of complex sequences between disparate neural populations brain wide, could be based on communications theory principles, especially using Single Sidebands (SSB). We show how these could be mediated by oscillations and nonlinear neuronal mixing mechanisms (Section 7). Strategies for testing the neural holography theory and the oscillatory cascade model are outlined in Section 8.

### 1.2 Information flow: how the topics are interrelated

A review of the literature turns up many papers linking together aspects of two or sometimes three of these topics (e.g., holography and associative memory, oscillations and phase-locking). This paper aims to propose a unified theory that links these topics together in a consistent, coherent framework. More background and detail for each of these is available in our recent publications (Cariani and Baker, 2022; Baker and Cariani, 2025; Cariani and Baker, 2026).

Many of the individual core ideas integrated into the unified neural theory proposed here have previously been expressed, researched, and tested to varying degrees, often decades ago by smart, insightful scientists. A number of these are referenced in the discussions below for neural coding and signal operations, traveling waves/oscillations, holography, communication theory, and related topics. In many cases their ideas were not pursued (e.g., high temporal resolution spike interval/phase-locking analysis, traveling wave functions, neural holographic models, etc.), due to technical limitations, and a failure to excite leading proponents of alternative research directions. Consequently, many of these ideas were not substantially developed, nor were they integrated into a consistent, coherent neural framework. Such a holistic framework is required to explain, relate, and test all the mechanistic stages by which the signals and stimuli received by the brain are converted into its functions, e.g., perception, motor action, cognition, executive decision making, etc. Nonetheless, empirical evidence consistent with these seminal ideas, individually and in limited combinations, has steadily grown over the course of time and technical advancement. The dedicated pioneers of these ideas, research, and technologies have laid the groundwork on which our theory and hypotheses are predicated/based. We owe them a debt of gratitude.

Intriguingly in 1958, Eccles foreshadowed some of the key concepts presented here (our interpretations added in parentheses and italics). He writes of the “working of the brain as a patterned activation formed by curving and looping of wavefronts (i.e., traveling waves), through a multitude of neurons, now sprouting (diverging), now coalescing (converging) with other wavefronts, now reverberating (recurrent networks), through the same path (reactivation)- all with a speed deriving from the millisecond relay time of the individual neuron, the whole wavefront advancing through 1 million neurons in a second”.

“Interaction of wavefronts (holographic interference patterns) originating at different sites in the cortex effects the integration and synthesis of the information relayed from different sensory receptors”. And further, “That the new evolving spatiotemporal pattern must tend to correspond closely (coherent pattern match) to the old, congealed (consolidated) pattern (memory trace); the impulses of the new pattern flow into a channel of the old and trigger (reactivate) its replaying.” (Eccles, 1958).

In the same paper, Eccles also proposed neuronal mechanisms corresponding to spike-timing-dependent plasticity (STDP), adaptation, conditioning, signal multiplexing, neuronal mixing, associative networks, and more.

## 2 Signal-centric neural architectures

The human brain is an extraordinarily powerful and complex general-purpose organ. Its organization and parallel processing enable it to process exogenous and endogenous signals, and to efficiently execute dozens of functions, learning new ones and adaptively revising previously learned ones. It operates in real time, and at low power, about 20 Watts, comparable to a low-wattage light bulb. While occupying only about 2% of body mass, it consumes about 20% of the body’s metabolic energy (Padamsey and Rochefort, 2023). The fact that the brain can process all it does on such low wattage is a tribute to its very efficient computations, an issue highly relevant to contemporary artificial neural networks.

The brain has been estimated to contain on the order of 100 billion neurons, each of which may connect to hundreds or thousands of other neurons. This dense, massively parallel architecture is characterized by extensive cross-connections that support traveling waves and reciprocal connectivities. These form ubiquitous recurrent local and global loops that support bi-directional oscillatory couplings. There are several means by which traveling waves and recurrent excitations can travel: via axons, dendro-dendritic, ephaptic couplings, and electrical fields. Neuronal components, from fine dendritic fibers to neurons and axons of variable cell types, are organized into diverse networks that coordinate their operations.

Different propagation speeds arise from volleys of spikes coursing through myelinated fiber tracts (fast) and unmyelinated fiber tracts (slower), as well as ionic flows through gap junctions and by volume conductions. Systematic differences in conduction speeds and their associated delays can potentially support spike latency codes (Cariani and Baker, 2022; Baker and Cariani, 2025). The relative delay between faster and slower waves can indicate specific classes of stimuli (e.g., auditory, visual, tactile). Here the different kinds of information can produce characteristic relative latencies of responses by responding populations (Cariani and Baker, 2025). A recent review article examines three decades of theoretical and experimental research relating spike timing for neural codes across multiple timescales (milliseconds for sensory coding/perception to hundreds of milliseconds to seconds for decision making). These spike codes underlie information coding and behavior (Panzeri et al., 2026).

As McCulloch pointed out (McCulloch, 1946), the neural architecture is heterarchical, rather than simply hierarchically organized, as is often portrayed. This complex, parallel architecture enables the brain to collect information about external and internal events, to process it in real time, and to store it for future use in support of the brain’s many current functions and future behaviors.

Traveling waves of activity broadcast information widely through a heavily interconnected network. When multiple representations of given signals converge and are integrated, a winner-takes-all principle can provide protection against noise in individual representations, providing a form of self-correction, thereby maintaining the integrity of signals. The network’s highly parallel, distributed design provides redundancy for noise robustness and error correction. Reentrant, recurrent loops of many types support feedback, working/persistent memory, memory recall/replay, hippocampal replay, associative networks. Extending his “tidal wave” cerebellar timing theory to the cerebral cortex, Braitenberg et al. theorized that this array of massively parallel recurrent fibers with its delay paths could detect coincidences and readily code/decode sequences of events (Braitenberg et al., 1997).

Closed loops are also formed through reciprocal connections. These provide redundant feedback operations (e.g., action, reaction), convergent networks enabling coincidence detection and signal integration, as well as recurrent network processes (e.g., echoic memory, working memory, etc.). Relative to their strength, saliency, etc., these closed loops continue to circulate and refresh specific signals and signal patterns.

The matrix of neural tissue is characterized by neuronal structures that operate at multiple scales to achieve local and global activations and communications. Although there has historically been a major emphasis on somatic (axonal) spiking activity, there is strong evidence for largely separate dendritic and axonal activity that processes signals in different, and potentially major, ways.

Relating dendritic activity to traveling waves, Eccles observed “…that although we could only examine synapses-junctions between brain cells-one by one, the branching of large fibers into fine fibers just as they are approaching a synapse makes it necessary for us to regard the electrical activity occurring in these fine fibered branches as forming a wavefront…” (Eccles, 1958).

In describing this “dendritic web” and its electrical activity in the visual system, Pribram (2013) asserted “…thus everything we see is first processed by the fine fibers of the retina before any nerve impulses are actually generated…” He further noted that the “electrical activity of the dendritic membranes oscillate between excitation and inhibition, such that locally dendritic [activity] occurs …forming patches of synchronous oscillatory activity” (Pribram, 2013).

Looking back, Pribram recalled Eccles much earlier suggestion that:

“Although we could only examine synapses-junctions between brain cells-one by one, the branching of large fiber axons into fine fibers just as they are approaching a synapse makes it necessary for us to regard the electrical activity occurring in these fine fibered branches as forming a wavefront….”

Pribram noted:

“I immediately recognized that such wave fronts when reaching synapses from different directions, would provide exactly those long-sought-after interference patterns Lashley had proposed… I also felt like an utter fool. The answer to my years of discussion about how such interference patterns among micro-waves could be generated in the brain had all the time been staring us both in the face-and neither of us had the wit to see it.” (Pribram, 2013).

In more recent studies, Moore and co-workers using sub-millisecond temporal resolution, have concluded that dendritic action potentials occur at several times the rate of somatic spikes, and that these vary over a wide dynamic range in a graded fashion as a function of the dendritic membrane potential (Moore et al., 2017). Dendritic action potentials, dendritic membrane potentials, and somatic spikes can be easily separately characterized and differentiated. The waveforms observed in each of these categories are non-sinusoidal and distinctively different from each other. They suggest that these and other characteristics can render dendritic branches “with branch-specific plasticity” into powerful computational entities, which integrate temporal and spectral information, largely separable from somatic activities. It appears that further exploring the roles of somatic and dendritic spikes and their interactions could be very helpful in understanding how the brain processes information, especially under dynamic conditions.

### 2.1 Convergent-divergent network organization

As is readily apparent from Ramon y Cajal’s elegant drawings to today’s electron microscope images, neural networks typically exhibit processes that broadly branch and *diverge*, fanning out and broadly distributing signals toward multiple targets. The signals themselves may thereby be broadcast or selectively propagated through diverse synaptic/network pathways, both locally and globally. Such processes are often observed to subsequently fan in, or *converge* on common synaptic targets that are presumed to integrate their respective communications, over time and space.

To the degree that the same signal or pattern is propagated simultaneously over two or more branches, the network operates like a holographic optical beam splitter. These two or more beams are analogous to coherent holographic reference and object beams. The neural tissue itself serves as a holographic medium at subsequent convergence points. Such tissue, embodied as synapses, neural ensembles, or dendritic networks, may record new interaction (interference) patterns or reconstruct previous interference patterns. At such points, auto and cross-correlation processes may serve as coincidence detectors for effectively recognizing previous object/memory traces in the network, thereby reactivating them, analogously to a holographic object reconstruction process.

The divergent-convergent design of neural networks could thereby implement holographic-like operations. When a spike train previously experienced, appears again, it may be processed/reactivated through similar pathways by similar cell ensembles and time-delay characteristics. Such activations further select, reactivate, reinforce, and modify those pathways. Through plasticity, new signals, can define and establish new idiosyncratic patterns and pathways *de novo*, without recourse to previous learning or training of them, e.g., 1-shot learning. The system is signal-centric in that the signals themselves are instrumental in self-organizing, modifying, and shaping the networks through which they propagate and interact. In short, the brain’s architecture with its wide distribution of signals through diverse interconnected recurrent networks, appears amenable to the kinds of dynamic signal processing suggested here.

## 3 Traveling waves and wave dynamics

Waves are fundamental. These can be external to the brain or internally generated. They define our world and how we see it, and feel it, and touch it. These continuous analog signals create our reality, how we think about it, what we do, and what we have done to us!

The brain is filled with waves of electrical activity on multiple scales, from subthreshold membrane potentials to large scale network activations. A traveling wave is a pattern of electrical activity that propagates from one brain area to another (i.e., spreading activation) as denoted by its changing phase in time and space. By contrast, a standing wave oscillates in time but is not a traveling wave in that it does not propagate spatially (Alamia and VanRullen, 2024; Cruddas et al., 2026).

Different stimulus-dependent patterns of spreading activation are widely observed in the brain (e.g., Marinković et al., 2003). Traveling waves of synchronized neural spiking activity spreading across the cortex are commonly observed as oscillations or brainwaves. The synchronized activity, primarily from ionic synaptic currents in large numbers of neuronal dendrites, comprise such oscillations. They modulate, and are modulated by, the diverse neural populations they encounter and interact with as they propagate. Subthreshold membrane voltages and oscillations that do not produce spikes themselves contribute to these. As observed with EEG/MEG, ECoG, and other studies, different frequency ranges of oscillations closely correlate with specific sensory, motor, memory, cognitive, sleep, and other functional brain states.

A recent review by Cruddas et al. takes an integrative multidisciplinary perspective in examining how the theoretical framework of physical wave dynamics plays a fundamental role in the neurophysiological mechanisms of cortical wave dynamics. Taking advantage of the new opportunities and spatial resolution afforded by dense cortical recording arrays, they analyze the linear and nonlinear nature of dynamic cortical waves, how cortical waves emerge, coordinate hierarchical operations, and encode perceptual and behavioral information across both mesoscopic and macroscopic scales (Cruddas et al., 2026).

Traveling waves sequentially activate multiple cell populations (both excitatory and inhibitory) in the course of their propagation. Through this sequential propagation reinforced by successive waves, they can incorporate recent history of their interactions. They can widely distribute information with high redundancy, ensuring noise robustness through increased signal/noise ratios, error correction, etc., and thereby maintain signal integrity/fidelity. Interactions between multiple waves of the same and different frequencies can result in both linear operations (e.g., constructive/destructive interference) and nonlinear operations (e.g., multiplicative signal mixing).

Traveling waves can broadcast arbitrary spatiotemporal/population patterns of activation across the brain. These waves create patterns of differential spike latencies that can support spike latency codes. Spike latency codes are the temporal correlations between spikes that indicate which subpopulations of neurons are firing by virtue of their latency relative to onsets, offsets, and other transients.

### 3.1 Oscillations support many functions

Many oscillations are generated throughout the brain and are associated with many different functions and behaviors. For example, in a faint visual target perception task by awake behaving trained marmosets, Davis et al. observed that spontaneous traveling waves improved target detection and sensitivity (Davis et al., 2020). Furthermore, individual frequencies can be correlated with different roles in different contexts. Weiss and Mueller cite multiple functions for beta processing within language processing (e.g., discrimination of word categories, semantic retrieval, syntactic binding, cell assembly organization/maintenance of short-term memory, novelty detection, language comprehension and motor control) (Weiss and Mueller, 2012). Beta has also been associated with top-down inhibitory control, especially for motor functions (Rossiter et al., 2014), as well as for cognitive processes (Lundqvist et al., 2024). Multiple oscillations within a given frequency band can even occur in the same region (Goyal et al., 2020). Different regional and task-specific patterns of brief beta have been observed in the striatum and motor cortex of monkeys engaged in the post-performance of reaching tasks (Feingold et al., 2015). They further report that, as compared with healthy human subjects, Parkinson’s patients (off medication) exhibit prolonged beta activity that may interfere with other neural processes.

Brain waves generally, and oscillations specifically, are presumed by many to mediate different, and likely multiple, functional roles (Kopell et al., 2010). They operate across a number of diverse separate and integrated networks. Some key mechanisms involving these, including synchronizations, phase coupling/coherence, patterns, etc., are discussed in Section 6.

Subsequent to their initial discovery in humans by Hans Berger (Berger, 1929), alpha waves (freq. Range 8–12 Hz) and beta waves (12–30 Hz), are now recognized as part of a larger set of commonly observed higher and lower oscillation frequency bands including gamma (35–80 Hz), high gamma (80–150 Hz), very high gamma (>150 Hz), theta (4–8 Hz), delta (0.5–4 Hz), and low delta (<0.5 Hz), as well as subbands within some of these.

The neuroscience research community have created a vast research literature to characterize different varieties of oscillations, explore how, why, and where they are generated, their propagation, their recurrence, synchronizations, cross frequency interactions and modulations, and to correlate them with diverse types of neural functions and behaviors. As is readily evident from visual inspection, physiological oscillations can vary substantially between stable and transient states. Increasingly, the generation, dynamic interactions, and possible roles of oscillations are being examined ever more closely (e.g., Buzsáki, 2006; Muller et al., 2018).

In their review of neural oscillations as causal or epiphenomenal, Van Bree et al. propose that oscillations “enable functional multiplexing,” and are central to the self-organization of “information processing within and across networks in the brain” (van Bree et al., 2025). Contemporary physiological oscillation research is conducted with ever more powerful tools and technology (e.g., microelectrode arrays). Some advanced computational models and artificial neural networks aim at emulating biological functions (e.g., image recognition). Others focus on trying to understand and relate theory to biological neural activity.

In exploring a computational model of analog neural oscillations, Singer and Effenberger (Singer and Effenberger, 2025) simulated a recurrent neural network with damped harmonic oscillators to demonstrate better performance on an unsupervised correlation-based digit recognition task based on MNIST. The Modified National Institute of Science and Technology (MNIST) handwritten digit database is commonly used for testing image processing systems. In another computational model, Jacobs et al., artificially produced traveling waves with a convolutional recurrent neural network to explore their potential role/benefits in effectively transferring and integrating spatial/visual information (Jacobs et al., 2025, preprint). Also applying a recurrent neural network with traveling waves to a simple image segmentation task, Liboni et al. report finding an exact mathematical solution, thereby enabling examination/explainability of their network’s visual computations (Liboni et al., 2025). Additional benefits ascribed to traveling waves used in conjunction with recurrent neural networks are obtained in robust spatiotemporal encoding and short-term predictions of visual sequences by recurrent networks (Benigno et al., 2023; Yiling et al., 2023; Busch et al., 2024).

Amid the ever-growing biological research on oscillations, their roles, their interactions, etc., Lundqvist and Wutz emphasize the substantial trend in recent years to move beyond static views of oscillations to much more time-varying dynamic perspectives, spawning new tools and approaches, and better modeling of the dynamic nature of brain functions and behaviors (Lundqvist and Wutz, 2022). Such research focuses both on the characteristics of traveling waves, and their relationships to function and behavior from sensory modalities to cognitive activities. These include for example, relating instantaneous frequency and phase of neural oscillations to power modulations and precisely timed bursting pattern activity for cognitive processes, consistent with many of the ideas presented with our temporal perspective.

In 2006, Canolty et al. reported transient phase-locking between high gamma oscillation power and theta oscillations, and distinctive cortex-wide patterns of these evoked by different behavioral tasks (Canolty et al., 2006). They suggest this mechanism can provide for communication in cognition processing.

### 3.2 Oscillations and their directionality

Asserting that oscillations are indeed traveling waves across the cortex Bhattacharya et al., explored how the directionality of the waves in different frequency ranges can shift as a function of working memory task performance, across the hippocampus, and other specific brain regions (Bhattacharya et al., 2022). They observed that traveling waves (theta, alpha, and beta) in the prefrontal cortex, consistently changed direction, including rotating during the course of monkeys performing a delayed match-to-sample working memory task. They suggest that the dynamics of these may play a fundamental role in predictive coding, sustaining memory, neurally tracking time through temporal phase encoding, inducing plasticity, etc.

Based on their human audio-visual intracranial delayed match-to-sample studies, Mohanta, et al. have suggested that traveling waves may carry predictive information, as well as mismatch signals for learning, as evidenced by alpha phase resets in conjunction with alpha-high gamma coupling (75 Hz-140 Hz), in the event of prediction errors (Mohanta et al., 2024). This led to posterior to anterior alpha propagation. However they observed that in an AV match-to-sample task, without signal mismatches, that stronger predictions were correlated with alpha band anterior to posterior traveling waves.

Earlier studies comparing hierarchical simulation models to EEG studies in visual cortex have also proposed that observed bidirectional traveling alpha waves could be involved in predictive coding (Alamia and VanRullen, 2019).

Mohan et al. have demonstrated that traveling waves can propagate in distinctively different directions as a function of separate cognitive processes and behaviors (Mohan et al., 2024). More specifically, they found that for episodic memory, the traveling waves they observed for the theta-alpha band (2–13 Hz), propagated in a posterior-to-anterior direction during successful memory encoding, whereas for recall, the theta-alpha band propagated in an anterior-to-posterior direction. They suggest that information may be encoded in packets of spikes (see section 7.3) that are propagated between contiguous areas on the basis of phase coherence in the traveling waves.

In their studies of sleep spindles, Muller et al. observe that with millisecond precision, spatiotemporal cortical spindle patterns are recurrently circulated through multiple brain regions by rotating traveling waves, up to hundreds of times per night (Muller et al., 2016). They propose that combined with synaptic plasticity, this mechanism could serve to help to refresh and reinforce networks for integrating and storing memories across the cortex. Recent work describes the spatial patterns (e.g., planar, concentric, rotational, complex spiral waves) and directionality of traveling waves correlated with behavior, e.g., (Das et al., 2018; Zhang et al., 2018; Das et al., 2024). This work and related research strongly suggests that in addition to studying which oscillation frequencies are observed, that the directionality of those waves must be determined as well. This has far-reaching implications, with respect to selectivity in routing, synchronizations, etc.

### 3.3 Neural manifolds

Activity patterns in the neural tissue of the brain can be characterized in terms of neural manifolds (e.g., continuous sheets, neural field theories (NFTs), and mean-field models) See (Deco et al., 2008; Breakspear, 2017; Zhang et al., 2023; Cruddas et al., 2026) for reviews. Neural manifolds (including slow manifolds) are analogous to mathematical manifolds (e.g., smooth surfaces with locally Euclidean geometry). These can be used to model the collective dynamics of coordinated population-based brain-wide neural activity, e.g., working memory functions (Ghazizadeh and Ching, 2021) as well as traveling waves and emergent oscillations (Wallace et al., 2011).

It should be noted that mean field models typically deal with averaged neural activity patterns taking place over much longer integration windows (e.g., fMRI data with sampling periods on the order of 700 ms or longer) than those related to the faster neural response patterns related to oscillatory dynamics, temporal codes, down to millisecond-precision neural processes.

To simplify the analysis of high dimensional neural data (the broad collection of individual neuron spiking observations), the dynamics of coordinated neural population activity are routinely analyzed with lower dimensional representations, such as principal components analysis. Such representations have sometimes been referred to as low dimensional “neural manifolds” and are being applied to traveling waves (Langdon et al., 2023; Mitchell-Heggs et al., 2023; Zhang et al., 2023; Kuzmina et al., 2024; Orepic et al., 2024; Perich et al., 2025; Shervani-Tabar et al., 2026). This framework represents a reduction of high dimensional input in terms of lower dimensional sparser representations.

Wave propagations need not depend solely on spikes. To the extent that neurons can partially be driven by common subthreshold oscillations that are induced by local or population-wide oscillations, the spike activity they produce could robustly synchronize to each other without requiring direct neuron-to-neuron synaptic interactions or connectivity. Traveling waves can produce synchronized spiking that can be used to organize and couple different neural populations. Similar proposals involving phase-locking to common underlying oscillatory potentials in olfaction have also been made (Brody and Hopfield, 2003). Loffler and Bahmer have proposed a computational model where subthreshold traveling waves of membrane potentials coordinate temporal and spatial codes (Löffler and Bahmer, 2026).

## 4 Holographic principles

How might holography be relevant to brain function? In many diverse ways the capabilities of brains resemble those of holographic representations. First, holographic techniques provide examples of how information can be represented, stored, and retrieved in a highly redundant, robust, and distributed fashion. Holography enables content addressability, pattern completion, and pattern similarity comparisons. Neural correlates of these functional characteristics are seen in hippocampal replay and pattern completion.

Holography relies on creating signal interference patterns, storing them in a distributed way, and recovering the signals from the stored form (“wavefront reconstruction”). Holograph-like processes may be implemented in neural systems using spatial and/or temporal interference patterns of interacting neural signals (e.g., spike trains, population oscillations). Particular stimuli produce characteristic temporal, spatial, and spatiotemporal spiking patterns (encoding). The interference patterns and their constituents may be read out by presenting networks with fragments of the whole pattern (decoding). Potential neural holography mechanisms based on spike correlation patterns, oscillations, and traveling waves are discussed in Section 4.5.

### 4.1 History of holography

While researching how to reduce optical aberrations in electron microscopes in 1948, Dennis Gabor discovered he could use Young’s principles of wave dynamics to record interference patterns from two beams of coherent (phase aligned) light, capturing amplitude and phase information of objects, on a photographic plate, a “hologram” (Gabor, 1948). When this plate was later illuminated, the original images could be seen again, a process he called “wavefront reconstruction.” Although recognized for this invention, Gabor seriously struggled with improving this process, until the advent of lasers in 1962. Suddenly much purer light coherence was available, and with that technology advance, holographic methodologies rapidly improved and practical applications were developed.

Key among these improvements was off-axis holography developed and popularized by Leith and Upatnieks which used a carrier frequency to separate the real and virtual images that had been superimposed in Gabor’s inline holograms (Leith and Upatnieks, 1962). In 1956, Lohmann had earlier proposed using single-sidebands for separating these two images, also drawing on communications theory, but his work was not well known or appreciated (Lohmann, 1956). See sections 6 and 7 for discussion of single sidebands, frequency carriers, brain analogs, etc.

Applications combining single sideband communications with holography have been demonstrated to improve image resolution and transmission efficiency (Ishizuka, 1994; Shoidin and Pazoev, 2021; Shoydin and Pazoev, 2021). Significant commercial and military applications; e.g. chirp radar and synthetic aperture radar (SAR) (Bryngdahl and Lohmann, 1968), etc., have also been developed on these principles.

New industries built on holography now span optical microscopy, computer-generated (digital) holography, interferometry, medical and industrial imaging, military applications, data storage/mining, weather predictions, etc. In 1971, Gabor was honored for his holographic discoveries with a Nobel Prize in Physics.

### 4.2 Encoding waveform interference patterns and reconstructing objects from them

A holographic process is the recording on a suitable medium (e.g., film) of the interference patterns of a coherent reference beam with an object beam, of which the subsequent activation of either will reveal the other. Typically, a re-exposure of the interference pattern on an optical hologram by the reference beam is used to reconstruct an image of the object. Holographic principles are directly derived from wave dynamics and wave interactions. Consequently they apply not only to electromagnetic waves (e.g., light, radio) but also to mechanical waves (e.g., water, sound/acoustic).

An interference pattern is essentially a standing wave pattern created by the interactions of two or more waves, momentarily stabilized. It captures the time, amplitude, and phase relations of the interacting waves, with high resolution. On optical holographic media, points of maximum constructive interference between the interacting waves, appear as white areas; points of maximum destructive interference appear as black areas. Given the multiple constructive and destructive wave interactions of the interacting waves, the interference pattern has high redundancy, and consequently, inherent error correction and robustness to noise. In optical holograms, the intersection points act like diffraction gratings. They capture multiple views of an object from many different angles, enabling the detailed recording of 3D visual objects onto holograms, which in turn, can be reconstructed and viewed from multiple angles.

Holography and the principles that drive it, are characterized by especially attractive and powerful properties. These enable broadly distributed informational representations of objects, events, attributes, etc. onto a recording/storage substrate (e.g., film), from which any piece can efficiently generate/recall the informational content of the original entity.

### 4.3 Neural holography

We conceive of neural holography as an organic self-organized process. Analogous to optical holograms, the neural substrate may produce and record interference patterns of excitations, inhibitions, subthreshold dendritic currents, spike trains, and traveling waves traversing the brain. Representations of these are subsequently consolidated in neural networks, both recurrent and non-recurrent, which driven by salience, attention, and other factors, may persist for varying durations. By their very dynamic nature, traveling waves and their interactions can widely distribute whatever information they bear.

We suggest that the neural substrate of the brain is similar in many respects to the holographic media or photographic film of optical holograms. The primary constituents of this substrate are densely packed neurons, comprised of cell bodies (soma), axons, and dendrites, and glia cells, e.g., astrocytes, microglia, oligodendrocytes. The highly divergent and convergent system of neural transmission patterns can potentially support reconstruction of previously acquired neural interference patterns.

There are some obvious significant differences between optical and neural holography. Although the brain does not have access to highly coherent beams of light, optical holographic operations can be effected without precise coherence (see discussion of white light holography below).

Possible neural interference patterns could be based on a variety of mechanisms that range from the dendritic webs mentioned by Eccles and Pribram above to spike correlation patterns to the interactions of oscillations and traveling waves. In the signal-centric perspectives, these are all interactions between patterned neuronal signals that have internal temporal, spatial, and spatiotemporal structure. The interactions are “signal dynamics” and the patterns arising from their interactions are “wave interference patterns” or patterns of intra-neural and inter-neural spike correlations.

How information encoded in neural interference patterns is recovered (e.g., wavefront reconstruction) is an open question. The theory suggests that if the neural interference patterns are initially formed with a specific set of oscillations and/or temporal patterns present, then re-presentation of those patterns could evoke reactivation (e.g., memory retrieval). There may be analogous neural operations in brains that iteratively collect fragments (see discussion of packets in Section 7.3).

#### 4.3.1 History of holographic brain theories

Although not labeled as “holographic,” theoretic models invoking holographic principles in the brain and its neural processes, are not new. They were largely built on the basis of waves, wave dynamics, and signal/mathematical processes combined with existing knowledge of brains, their physiology, neurodynamics, and behaviors.

As a consequence of his extensive lesioning and related research, Lashley proved that trained rats could lose much of their cortex without catastrophic ill effects. In 1929, he hypothesized that neural information and learning; e.g. memory representations, were encoded in widely distributed (interference-like) patterns reflected in spatiotemporal patterns of localized activations (Lashley, 1929). He further proposed “mass action” by which functions typically supported by specific brain regions, could be assumed by other brain regions in the event of damage to those specific areas (also see John, 1972).

Beurle (1956) used wave dynamics to theorize/explain how self-organized informational neural waves could be generated, interact to form interference patterns, be propagated and stored, and regenerated from memory. He realized that two traveling waves in the cortex could together generate an interference pattern, from which either wave could subsequently be used to regenerate the other. Along similar lines Heinz has proposed applying his time-domain based Wave Interference Network to both the brain and electronic communications (Heinz, 2004).

Roy pursued theories of associated learning and memory retrieval based on neural ensembles generating and storing automatically encoded informational patterns realized as signal correlations stored in variable delay line structures, including recurrent replay loops (Roy, 1960, 1962).

With the holography breakthroughs of the early 1960’s, came an immediate realization of even stronger analogies between physical holographic processes, and neural processes in the brain, especially associative memory processes. A volley of research and theoretical models followed.

Longuet-Higgins (1968a, 1968b) started looking for ways to translate Gabor’s frequency domain (Fourier based) optical holography methodology into a more neurally plausible time-domain methodology. He theorized a mixed strategy using a filter bank of narrow band resonators to record temporal signals, then proceeded to propose time-domain signal correlations instead of spectral analyses (Longuet-Higgins, 1968a; Longuet-Higgins, 1968b). He also proposed a Fourier-based theory of a temporal holograph called the “holophone.” In 1989, he described his evolved time-domain theory to show how holographic memory could be achieved using temporal correlations and convolutions of spike trains (Longuet-Higgins, 1989).

Gabor engaged in active ongoing discussions with Longuet-Higgens and others to improve their neural modeling (Gabor, 1968). Reported as saying that “it’s something like Fourier, but not quite,” Gabor proposed his own model using truncated correlograms and for coding and truncated convolutions. He aimed to model the computations involved in recalling temporal sequences from a fragment of memory, and continued to refine his mathematical theories for implementing holographic coding and recall (Gabor, 1969). With respect to brain holography, Van Heerden asserted that the foundation for it was essentially based on information theory as used in radio, television, etc. He invoked matched filter correlation functions and propagating wave fields (Van Heerden, 1970).

Other key players actively engaged in pursuing better holographic theories and correlation-based neural models include: Brindley, Marr, Pribram, Westlake, Cavanaugh, Chopping, Wilshaw, Barsellino, Poggio, Reichardt, Kohonen, Plate, Kohonen, Nickel, Hinton, Anderson, Carrillo-Reid and others: (Reichardt, 1957; Reichardt, 1961; Brindley, 1964; Chopping, 1968; Westlake, 1968; Brindley, 1969; Willshaw et al., 1969; Westlake, 1970; Pribram, 1971; Barsellino and Poggio, 1972; Cavanaugh, 1975; Kohonen, 1987; Hinton and Anderson, 1989; Marr, 1991; Plate, 2003; Pribram, 2013; Nickel et al., 2016; Carrillo-Reid et al., 2019; Pietsch, 1981).

Common factors uniting most of this work are the significant roles played by wave/wave dynamics, precise time/phase relationships, and analog computations using correlation/convolution mathematics to describe how signal interactions could generate, store, propagate, and recall information. For more background/detail on holography and the brain, see section 4 in (Baker and Cariani, 2025).

### 4.4 Self-organized Hebbian cell assemblies

Our theory proposes that neural interference patterns are initially instantiated as precise spike/spike train codes, which progressively evolve into specialized spatiotemporal patterns reflecting the successive neural processing stages they undergo.

These distinctive patterns could operate as temporal signatures for objects, concepts, events, etc. Traces of these are recorded and maintained recurrently in memory by neuronal elements. When these distinctive patterns (or subsets of them), are encountered on subsequent occasions, their traces can be reactivated through coincidence detection and correlation processes. Once reactivated, these pattern traces are reinforced. They become more excitable and trigger further activations. These include the recruitment of additional neurons into cell assemblies, for dynamic Hebbian “fire together, wire together” connectivity. Using STDP processes, such connections can be made transiently or persistently. New self-organized associative networks are thereby established. In this manner, associations between cell assemblies sharing common attributes consequently become linked in content-addressable networks.

That is, the associations themselves and the networks they share, define and establish the links or traces connecting each other. Arbitrary chains of these can be constructed or linked through the activation of common attributes. The synchronized activation of multiple associative networks can operate to clearly distinguish between competing choices. Winner-take-all processing principles determine final choices. Because the attributes and/or associations themselves can have different strengths and/or priors in different networks, alternative evaluations of these associations can be made. Associative networks and subsets of these, are thereby created and flexibly adapted with features added, subtracted, or modified. The attributes themselves can be bound by bottom-up characteristics, as well as bound probabilistically on the basis of past experience (e.g., priors). So although fire engines are usually red, one still can recognize a blue fire engine. The predictive preponderance of other fire engine attributes determine its identity. Having seen one blue fire engine, you increase your expectations, that you might see another.

The word “read” in text can be used as a noun or verb. Without semantic context, its meaning is unclear. In speech, the same phonetic string r-eh-d can be interpreted as the color red, or the word “read,” again depending on semantic context. Ambiguities are resolved based on signal (re)processing and the joint likelihood of specific attribute combinations in the context of activated associative networks.

Complexes of common associations (routinely associated features) can operate on different scales, incorporating more or less detail. The most detailed are like high resolution photographs. Visuals can be successively reduced or scaled down to sketches, or even cartoons. Gestalt images characterized by sparse relational representations, can be recognized invariantly, independently of size, color, texture, etc. (e.g., triangles). Johansson figures with only a few 2D joint markers (e.g., head, hip, shoulder, elbow, knee, foot), convincingly convey 3D biological motion for people and animals (Johansson, 1973). Invariances, including scale invariances, are exemplars of such feature sets. These can be modelled in associative networks where scale (or relational scale) is simply an attribute.

Separate complexes of consistently associated attributes help define and identify discrete objects, and distinguish them from one another. Backgrounds are characterized by (e.g., relegated to) low salience. They typically consist of stable, predictable, often slowly changing sets of objects or attributes held in bound complexes. By contrast, significant signal or stimuli discontinuities are typically detected by abrupt onsets/offsets of various types. Importantly, predictable background discontinuities, e.g., leaves quivering in the breeze, can be suppressed or masked while still being monitored. By contrast, unaccustomed or unexpected discontinuities (e.g., wind gusts suddenly cracking branches on a calm day) immediately trigger and focus attention (increased salience). Although such discontinuities may be loud, they need not be. An unexplained violation of expectations (predictions) is sufficient (e.g., an unusual sound).

## 5 Neural codes, representations, and signal-centric processing

The neural system is subject to a continuous stream of exogenous as well as endogenous signals. Some of these are dynamically changing; others are relatively stable steady state signals. From those signals the brain needs to be able to encode, propagate, retrieve, decode, and use whatever information is available to perform its many functions and behaviors (e.g., make and distinguish fine distinctions in sensory data, coordinate consistent motor actions, interpret and respond appropriately to cognitive, emotional, motivational demands, etc.). The brain requires a common framework with which it can process all the diverse signals and circumstances it encounters.

Spikes, spike trains, and patterns of these, constitute the internal signals of neural systems for vertebrates. This can also potentially include neural response patterns on ensemble and population levels. These encode stimuli, and representations in subsequent neural processing stages. Both temporal coding and rate coding of sensory information are widely evident in their sensory systems (Cariani and Baker, 2025). Despite decades of research and much progress though, there are still large gaps in our knowledge of the coding and processing of spikes, especially at cortical levels.

External signals routinely processed by the brain encompass a wide variety of waveforms. Typically, sensory waveforms consist of a mix of steady states and dynamically changing states. For example, in raw (unprocessed, optionally sampled) acoustic speech waveforms, the vowels and nasals are typically characterized by repetitive pitch periods, whereas many consonants (e.g., stops) as well as many phone, syllable, and word boundaries are characterized by rapid transients and aperiodic states.

Waveforms, of all kinds, can be described as series of numerical amplitude values., i.e., a one-dimensional signal of amplitudes. Most often the series is a time-series of numerical amplitudes, such that the waveform is a description of a signal in the time domain. Discrete waveforms can be derived from continuous-valued analog waveforms by sampling them at discrete time intervals. For digital computer analyses, analog waveforms need to be sufficiently sampled and digitized. Typical digital sampling rates for acoustic-phonetic analyses in speech recognition range from 16 kHz to 44.1 kHz (audio CD fidelity). Higher audio sampling rates (e.g., 48 kHz and 96 kHz) are often used for voice transformations/manipulations and artifact removal in video production and high fidelity studio recording, as well as other kinds of speech audio analysis.

The frequency domain representation of a digitized waveform is a spectral decomposition reflecting its component sinusoidal frequencies and their amplitudes averaged across an integration window. Fourier analysis is typically used to calculate this transformation. For speech waveform analyses, typical integration window durations are 20 msec overlapped by 10 msec. Acoustic events lasting only a few msec are necessarily undetected or smeared. Such events are readily observed as sharp discontinuities in the digitized or undigitized waveform itself prior to such spectral processing. We contend that rapidly changing, dynamic signals need to be tracked with adequate temporal resolution for good characterization. Longer duration periodic signals can be well characterized by using standard integration windows.

### 5.1 Signal assumptions and analyses

Thorpe and colleagues have long argued for the necessity of neural temporal coding strategies based on observations that many behavioral responses are completed too quickly for the underlying sensory processes to rely on neural firing rates which are computed over tens to hundreds of milliseconds. More specifically they have asserted that typically fast visual recognition is inconsistent with firing rate codes and have proposed a number of temporal spike codes as an alternative (Gautrais and Thorpe, 1998; VanRullen and Thorpe, 2001; Thorpe et al., 2004). Also asserting the necessity of precision timing, Luo et al. have proposed concurrent transitioning frequency and amplitude modulation processes (Luo et al., 2007). In recording from populations of single units in the human anterior lobe during a visual categorization task, Xie et al. found that the information encoded in the temporal ordering of spikes (i.e., spike interval sequences) during spike bursts was independent of and complementary to single unit spike rates and latencies (Xie et al., 2024). That is, the combined information derived from these two analysis methodologies, yielded higher categorization performance, than using either alone.

Much of neuroscience research and its many fruitful advances have been based on such traditional frequency domain analyses. The theory proposed here posits that incorporating a complementary time domain perspective, may yield significant benefits (e.g., enabling precision temporal resolution for spikes, spike coding, multiplexing, phase-locking and coupling, coincidence detection, spatiotemporal patterns).

The first step for a researcher is to capture the waveform of the original stimulus with adequately high temporal resolution. High resolution temporal representations include phase-locking, spike interval analyses, etc. Averaging spike intervals by averaging or “binning” them, substantially reduces or destroys their inherent high temporal resolution, and their information content.

Average rate codes necessarily provide course/rough temporal approximations. In practice, they often average signals across fixed windows that are longer than critical events in the signal or system itself. For example, neural population responses are often processed with spectral integration windows of 10–20 ms even when meaningful/significant events are happening more rapidly, on the timescale of milliseconds or sub-milliseconds, an order of magnitude or more, faster. Though computationally convenient to calculate (e.g., using FFTs or other spectral decompositions), these time-averaged population measures can easily obscure and destroy significant signal information that cannot be subsequently recovered. For example, the averaging of brief spike bursts (e.g., at stimulus onsets) occurring within such windows can misleadingly be interpreted as indicating consistent steady states across the duration of those windows.

Similarly, the application of high and low pass filters, especially to rapidly changing signals, can create significant distortions. Filter banks are typically used to enhance or “sharpen” spectral components within a given frequency band. Although useful for many contexts, they impose spectral integration windows which can obscure and destroy fine temporal information. In the process of characterizing and analyzing signals, endogenous or exogenous, care must be taken to maintain and preserve their information content. In order to understand neural mechanisms, we need to apply suitable analytic methods to study and test them.

### 5.2 Temporal codes and signal-centric processing

Temporal codes represent information in terms of spike timings, and patterns of these. They are fundamental to a signal-centric perspective in that the successive generation, correlation, and interaction of spiking patterns, simple and complex, determine neural actions and behaviors. The simplest time code for spike sequences is simply the time series of interspike intervals. Information is encoded in the durations of time between spikes (e.g., pairwise, consecutive or not, etc.), within and between spike trains. Simple and complex patterns of these can be constructed, correlated, and multiplexed within and across modalities.

By contrast, rate codes (channel codes), average spike firing rates over a given time period (i.e., integration window) to represent information. These have often been associated with particular rate-place channel activations. Temporal codes and rate codes are complementary and may be combined.

Detailed descriptions of temporal codes, time-domain neural networks, spike coincidence mechanisms, signal-centric computations (correlations/convolutions), neural timing nets, and adaptive time-delay neural networks have previously been reviewed (Cariani and Baker, 2022; Baker and Cariani, 2025; Cariani and Baker, 2025; Cariani and Baker, 2026). Ensemble and population-wide collective neural responses, such as traveling waves/oscillations may play essential roles in neural coding (Hopfield, 1995; Cariani and Baker, 2022; Baker and Cariani, 2025; Cariani and Baker, 2025).

### 5.3 Phase-locking to waveforms and temporal correlations among spikes

Temporal phase-locking analysis provides a simple time-domain representation of waveforms. This analysis converts a continuous analog waveform into a temporal sequence of event markers (e.g., waveform zero-crossings). It typically incorporates amplitude information as well. Such phase-locking enables the detection and tracking of periodicities as well as discontinuities in arbitrary waveforms (speech, music, spike patterns, etc.) with high temporal resolution and fidelity, irrespective of their spectral composition. Any waveform, periodic or aperiodic, can be tracked by recording the series of times or time intervals, phase-consistently from one waveform cycle to the next; from peak amplitude-to-peak amplitude, or from up zero-crossing to up zero-crossing. This transformation substantially reduces the dimensionality from the waveform sampling rate, and is widely observed in neurons, especially for spike coding of sensory stimuli.

Despite this massive dimensionality reduction, phase-locking typically preserves much of the information in the original waveform, even for dynamically changing waveforms. This information preservation became evident with Licklider’s demonstration of the high intelligibility of infinitely peak-clipped speech in 1948 (Licklider and Pollack, 1948). Infinite peak-clipping phase-locks to the speech waveform. This simple cycle-by-cycle transformation efficiently runs in real-time on continuous waveforms and spike trains. Phase-locking has the benefit of simultaneously maintaining excellent temporal, amplitude and phase resolution.

In the early 1960’s, Lettvin designed his Continuous Log of Ongoing Events (CLOOGE) electronics to generate and record real-time phase-locking of neural signals for his sensory and CNS research (Chung et al., 1970; Chung et al., 1974). This windowless method avoids integration over temporal windows, preserving the sampling rate precision of the original signal. This same time-domain methodology was subsequently applied to speech waveforms for better characterizing them, especially for the detection of informational transients and signal discontinuities (e.g., distinctive phone/syllable segmentation) and to improve speech recognition (Baker et al., 1972; Baker, 1975; Baker, 1979).

An earlier electronic analog device, the “Correlatograph” was designed and constructed by Bennett in 1953 to overcome known deficiencies of the spectrograph, especially for processing speech (Bennett, 1953; Biddulph, 1954). It displays in real-time, a short-term plot with x and y axes displaying running time and lag time, and the pattern intensity representing the correlation function (Lange, 1967). Similar plots, called “autocorrelograms” were used to analyze neural short-term spike train interspike interval patterns in connection with the coding of pitch in the auditory nerve (Cariani and Delgutte, 1996).

Given today’s large neural array recording devices and sophisticated techniques, it could prove very informative and useful to record dynamic stimulus waveforms (e.g., continuous speech), analyze them with high temporal resolution, etc., and then correlate patterns of these to simultaneously recorded neural spiking activity which has been similarly phase-locked. It would be especially interesting to correlate intralaminar and other ECoG data from multiple anatomical regions along the speech/language cortical hierarchy. Raw waveforms must be suitably recorded to maintain temporal resolution for high fidelity precision analyses. Comparisons should be made between speech listening, speaking, reading, etc. These high temporal resolution studies can then be compared with other conventional analyses as well.

### 5.4 Precision timing is observed across processing levels

The presence and significance of precision timing at sub-millisecond and millisecond levels for both transient event onsets and sustained periodic structure, is well established in early stations of ascending sensory pathways. There is a preponderance of evidence for temporal precision of neural responses across sensory modalities (Mountcastle, 1967; Perkell and Bullock, 1968; Carr, 1993; Cariani and Baker, 2025), especially associated with stimulus onset times. Despite many fewer studies, Singer reports, as have others, that precision timing is also observed at multiple cortical levels (Singer, 1999; Mackevicius et al., 2012). These studies demonstrate that neurons, even over several polysynaptic stages, can maintain millisecond precision (Phillips, 1993; Livingstone, 1996; Heil and Irvine, 1997; Buracas et al., 1998; Friedman-Hill et al., 2000).

Synchronization of discharges can coordinate interacting populations, establishing common precision timing. Phase-locking to these, can create new codes, population patterns, and more synchronized couplings. This process may be progressively iterated at each stage, thereby not only maintaining, but, depending on excitatory/inhibitory gating, possibly even increasing temporal synchrony, power, and coordination.

The neural system necessarily processes and integrates temporal and spectral characteristics of incoming signals. These processes proceed in tandem and are well suited for signals characterized by a mix of dynamic and steady states. As with other dynamic events, temporal processing best detects and characterizes sharp or sudden state transitions, i.e., stimulus onsets. Frequency domain processing and spectral analyses work best on periodic, steady state (slowly changing) signals. The neural system routinely processes complex combinations of both.

It is observed that the synchronization and couplings of oscillations across diverse frequency bands, and even within any single frequency band, are predicated on temporally driven neural processing principles (see Section 6). These critically depend on phase coincidence and coherence for their coordination and effectiveness. The alignment of phase and coherence between two or more oscillations is temporally precise. Timing matters not just for determining spike codes but for the coupling and integration of signals at all processing stages. Based on MEG/EEG studies for a visual working memory task, Haque et al. identified long-range, alpha-band phase synchronization networks (Haque et al., 2025). These networks supported and maintained neural response patterns related to different visual features (shape, color, location) as well as shared memory functions.

As signals progress up the cognitive hierarchy, it is observed that lower stages (e.g., sensory inputs) are associated with higher frequency oscillations (e.g., gamma), and that higher cognitive processing stages (e.g., feedback, concepts, events, decisions) are associated with lower frequency oscillations (e.g., theta). Bottom-up gamma modulated by top-down alpha/beta are commonly observed. Integrations and couplings of a variety of feed forward, feedback, lateral, and recurrent signals occur as traveling waves propagate across the brain’s networks. These integrations and couplings may well occur as detectable transient events.

There has been much debate and theorizing as to how, if, and to what degree, temporal codes are transformed to rate codes. It has often been conventionally assumed that temporal codes are routinely converted to rate-channel codes of one sort or another. Many neuroscientists have theorized that as signals are progressively processed, grouped, and categorized into stable states, that the use of averaged firing rates may be most appropriate for characterizing them. Others have argued that cortical neurons are better characterized as coincidence detectors rather than rate integrators (Abeles, 1982b; Abeles, 1982a, 2003). Ahissar (1998), and Ahissar et al., 2023 has suggested that phase-locked temporal codes in somatosensory peripheries are converted to central rate codes at cortical levels using phase-locked loops (PLLs); e.g. to convert sensory temporal signals to cortical oscillations (Ahissar, 1998; Ahissar et al., 2023). There is clear evidence of stable states being well represented by rate codes. But given the observations of precision timing at cortical levels though, it appears that the full story may be somewhat more complex (Luczak et al., 2015).

Our theory immediately suggests two (complementary) hypotheses for preserving and/or establishing temporal precision at high cortical levels. First, is that the brain often responds vigorously to signal discontinuities such as stimuli onsets, offsets, and expectation/prediction violations in the form of Mismatch Negativities (MMNs). These are signature events that designate state changes (state boundaries, onsets), and may be used and preserved as essential temporal referents, etc.

Second, the timing of traveling wave interactions is inherently exact. Whatever their respective frequencies, velocities, functional associations, (dis)similarities, processing stages, etc., whenever two or more waves interact, that interaction necessarily occurs at a discrete (i.e., precise) point in time. The sudden onset of wavefront interactions between traveling waves, and the information carried by them, whether instantiated as spatiotemporal patterns, bursts, packets, etc., constitute discontinuities that can also serve as time referents. Determining whether these interaction event times are significant or epiphenomenal, remains to be seen, of course!

Whether or not these frequencies linearly add or combine non-linearly (e.g., multiply), substantial discontinuities in amplitude/power can occur (See Section 6.6). It is not surprising that these discontinuities are observable at cortical levels, especially since discontinuities can trigger onset responses, spikes, bursts, etc. Recently Sejnowski has proposed that precision spike-timing in cortical neurons, which is stimulated by traveling waves, could instigate STDP as well as long-term working memory recall (Sejnowski, 2026, preprint). In their recent review article of spike timing timescales, Panzeri et al., conclude that “precise spike timing is an important part of the neural code meant as a unit of information used in the entire processing chain from emission to encoding to generation of behavior “(Panzeri et al., 2026).

## 6 Neural signal processing (synchronies, coherences, mixing, coupling)

The understanding of basic biological processes for how, when, and where the signals of the brain process information to perform its many functions and behaviors, is important. Its ramifications span health maintenance and restoration, disease prevention, medical interventions, and a range of technological innovations. This section reviews some of what we think we know about neural processing principles and how these might be organized and related to each other, spatially and temporally.

The Hebbian *“fire together, wire together”* principle by which the brain largely self-organizes, is generated by the triggering of active (firing) neurons or neuronal assemblies to activate (connect) with others, and subsequently to be synchronously coactivated, in response to the *signals* they receive (Lashley, 1929; Hebb, 1949; Nadel and Maurer, 2020). That is, the signals themselves, both excitatory and inhibitory, are instrumental in defining and refining the network topographies they traverse. Neural plasticity enables synapses and couplings between these elements to be created, adapted, and reinforced.

With reactivation, these connections can be selectively strengthened and extended. These mechanisms support adaptive learning, memory, and synaptic potentiation/inhibition. With successive activations, larger, more specialized and more widely distributed recurrent and associative networks can be grown. From these processes we observe a wide array of brain characteristics, from “one-shot learning” to habit formation. Successive iterations of distinct networks and their interactions thereby get associated together. Their coactivations enable yet higher level functions, such as multimodal sensory integration, chunking, delayed memory recall, and ultimately communication and coordination across the brain, culminating in perception, motor action, decision making, and cognition.

Through synaptic connections, Hebbian mechanisms bind similarly active neurons or groups of neurons together. Common firing patterns of activation enable neuronal elements to form and grow larger neural ensembles. Through synaptic time dependent plasticity (STDP), these ensembles act collectively, to form new, and reinforce previously established pathways. This process of establishing and engaging selective associations of neurons and their ensemble networks, form the basis of learning and memory capabilities; that is, how one thing is similar or dissimilar to another.

The sequences of cell assembly activations (rat hippocampus) are similarly bound (Almeida-Filho et al., 2014). Associations between memories and their components (e.g., people, places, attributes, objects, concepts, actions, temporal relationships) are based on their common contents; that is, by what they have in common or by contrast, with each other. Consequently, memories themselves are content-addressable. Recalling a specific memory activates a diverse set of related associations through recurrent networks. These can be closely related and easily accessed, or much more distantly/weakly, through chains of associations (e.g., enabling delayed recall). Activating these content-addressable association networks consequently evokes and predicts larger patterns (e.g., pattern completion) and likely events. Analogously to holographic interference patterns, the informational content stored in association networks is broadly, though non-uniformly, distributed.

Although they can be longer lasting (e.g., recurrent memories), interactions between ensembles and networks can be, and necessarily often are, very transient for making and breaking connections, locally and long-range (Biel et al., 2021). Individual neurons and neuronal ensembles may engage in multiple cell populations. The operations they perform are not tied to specific neural elements (“labeled lines”) *per se*. Redundancy, efficiency, and robustness against damage is thereby intrinsically achieved and maintained. The high redundancy of parallel processing can also provide for built-in error correction, through competitive processes whereby the preponderance of neural evidence (e.g., winner-takes-all) prevails even amid conflicting evidence.

Several key synchronization mechanisms have been proposed and are broadly believed to coordinate neural signals for association and communication locally and globally across the brain. Among these are coincidence detection, binding by synchrony (BBS), communication through coherence (CTC), and cross frequency coupling (CFC). To these can be added, binding through common temporal patterning (e.g., via temporal/spatiotemporal pattern processing (STPP)) which can exist between the spike patterns of individual neurons and local ensembles to oscillatory patterns of whole populations. These processes operate at a variety of different time scales, starting at sub-millisecond resolution, and can instigate STDP, critical for learning, memory, and adaptation.

Mechanisms for coordinating and coupling signals, require common temporal and phase relationships. Temporal correlation (e.g., simultaneity, similarity) and/or phase coherence is typically used for combining, multiplexing, synchronizing and integrating signal information (e.g., bottom-up and top-down oscillation interactions) and for information transfer. Taking into account the balance of excitatory and inhibitory influences, these temporal/phase relationships determine how information is gated, routed, adapted, enhanced, and suppressed or masked. References relevant to these issues include: (Von der Malsburg, 1999; Singer, 1999; Varela et al., 2001; Buzsáki, 2006; Womelsdorf and Fries, 2006; Womelsdorf et al., 2007; Sauseng and Klimesch, 2008; Siegel et al., 2009; Canolty and Knight, 2010; Fell and Axmacher, 2011; Jirsa and Muller, 2013; Fries, 2015; Lowet et al., 2022).

The synchronization and coherence (simultaneous activation) of neurons and ensembles is observed through a variety of mechanisms, especially phase coherence. Communication through coherence is posited to be a primary means through which information is communicated through the brain’s neural networks across and between frequencies, e.g., (Fries, 2015). Cross frequency coupling (CFC) refers to interactions between different frequencies. These synchronization processes can be integrated (Gonzalez et al., 2020), especially in conjunction with phase-locking processes, for proposed cross cortical communications (Cariani and Baker, 2022; Baker and Cariani, 2025).

As an alternative means of causally synchronizing neural populations, Schneider et al. have also demonstrated that common spiking activity from separate afferents in a given area can create coherence patterns (Schneider et al., 2021). In ECoG microelectrode array task-related studies in human epilepsy patients, Myers et al. have examined spike and spike-field coherences in frontal, temporal, and occipital cortices. They observed that coherence spatially extends up to 3 cm around a given cortical area. The magnitude of alpha coherence (8–14 Hz) was higher than for other frequencies for signals near the microelectrode array, whereas the magnitude of delta coherence (1–3 Hz) was higher for signals more distant. In all regions, the action potentials were most phase coherent with alpha oscillations. This suggests to them that local alpha waves may affect local spike-timing to a greater degree than other frequencies (Myers et al., 2022).

### 6.1 Oscillatory coupling within and across frequency bands

In signal processing terms, the coupling of two systems can involve linear additions of signals (superpositions) or nonlinear multiplications (mixing). Thus “coupling” can consist of additions or multiplicative mixings of neural signals and/or traveling waves/oscillations. In the context of interacting neural populations between two regions, additions and multiplications can also co-occur if different neuronal subpopulations combine local and incoming oscillatory signals in different ways. Whereas additive interactions produce patterns of constructive and destructive interference, multiplicative interactions produce new frequencies (see Sections 6 & 7).

Although the designated borders of the canonical frequency bands are not precise, the existence of the bands (and subbands), and their major distinguishing features, are evident in a wealth of empirical observations. Typical mid-point frequencies for these bands are approximately: 180 Hz (gamma very high), 90 Hz (gamma high), 30–45 Hz (gamma), 21 Hz (beta), 11 Hz (alpha), 5 Hz (theta), 2.5 Hz (delta) 1.0 Hz (delta low). These exact values can and do shift under different conditions (e.g., stronger stimulation) and measurement methodologies. Nonetheless, it is readily observed that the relationship of these characteristic values for neighboring bands is about 2:1 (e.g., gamma:beta, beta:alpha, alpha:theta, theta:delta, etc.). That is, for neighboring bands, the mid frequency of the next higher frequency band is about twice the mid frequency of the next lower frequency band.

By this formulation, neighboring frequency bands exhibit an octave (2nd harmonic) relationship. Section 7 gives an explanation for how these 2:1 frequency band relationships might be emergently generated by multiplicative neural signal mixing interactions. Alternative hypotheses for generating neighboring frequency band relationships based on multiplicative factors, have been proposed by Kramer based on the Golden Ratio = 1.62 (Kramer, 2022), and by Penttonen et al. based on natural logarithms = 2.17 (Penttonen and Buzsáki, 2003).

### 6.2 Cross-frequency couplings (M:M, M:N)

Couplings between non-adjacent frequency bands are also routinely observed, e.g., theta (~4–8 Hz) and gamma (~30–45 Hz). Based on cross correlation measurements, pair-wise couplings between different specific frequency bands associated with different physiological states and functions, can vary widely (Chen et al., 2022). The functional roles played by specific frequency couplings are an area of active debate.

Preferred non-neighbor band couplings also reflect integer values (e.g., gamma:theta, about 8–10:1; alpha:delta 4:1). This is a consequence of the conditions imposed for oscillation coupling via synchrony, phase coherence/alignment, phase reset, etc. Such integer relationships also provide a general mechanism for hierarchical “nesting” (e.g., gamma in theta). Faster frequencies in such relationships with slower frequencies, are said to be nested within them, and may be modulated by them. Many couplings are observed to correspond with functional relationships as well: hippocampal interareal synchronization (Fujisawa and Buzsaki, 2011); local/global oscillation communication pathologies (Salimpour and Anderson, 2019); precise spatiotemporal firing during memory retrieval (Herman et al., 2013); and speech processing (Giraud and Poeppel, 2012).

**Figure 3:**
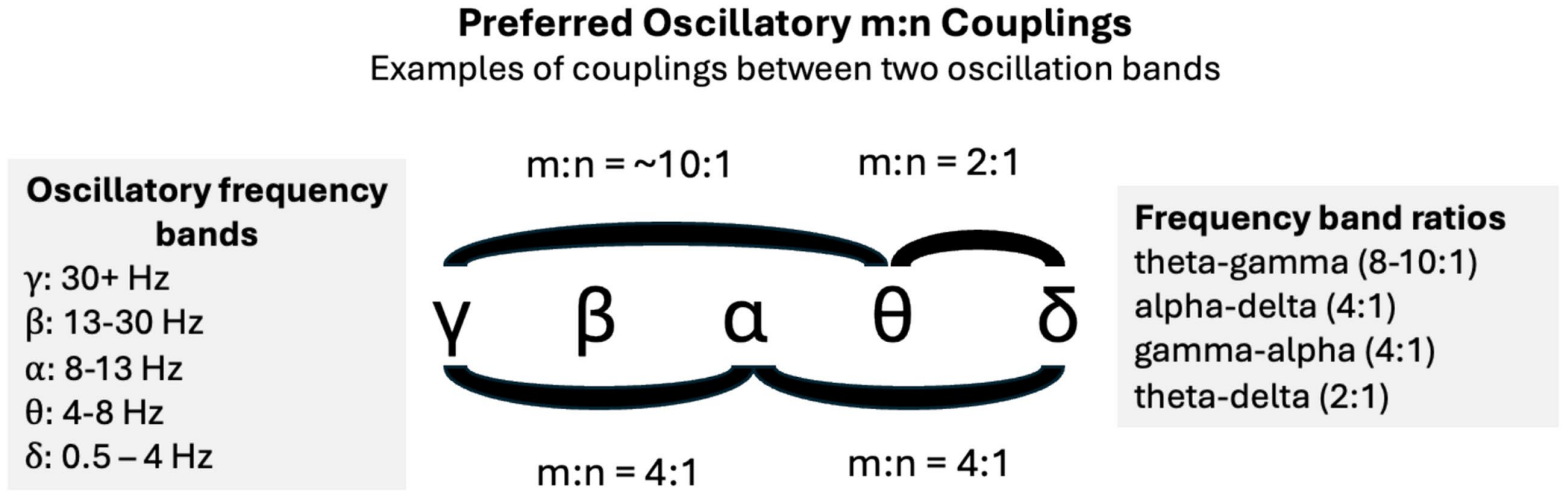
M: N oscillatory couplings.

M:M and M:N Cross Frequency Couplings (Figure 3) refer to couplings between separate oscillations at the same frequency (m:m) as contrasted with couplings between separate oscillations in different neighboring or non-non-neighboring frequency bands (m:n). When two oscillations of the same frequency are coupled, the 1:1 integer relationship between them is referred to as “m:m coupling.” This is consistent with a simple propagation of a signal across a synapse. When two oscillations with different frequencies are coupled, the relationship is known as “m:n coupling,” where m represents the faster frequency. This kind of coupling is consistent with oscillations from different sources being integrated together. Note that for both m:m and m:n couplings, the coupling/binding of higher frequencies (e.g., gamma), can produce more precision in temporal/phase resolution than is required for lower frequencies (e.g., theta), in synchronization and coupling processes.

M: N couplings regularly exhibit small integer relationships between interacting frequency bands. Exact frequencies are somewhat variable, and frequency bands are approximate ranges. However, it is observed that a low-end alpha band (8–12 Hz) is roughly twice as fast as a low-end theta band (4–8 Hz), expressed as a 2:1 m:n relationship. With a gamma of approx. 40–50 Hz and a theta of about 4 or 5 Hz, the m:n integer ratio is approx. 8–10:1. In such m:n relationships, the oscillation with the faster frequency is said to be “nested” within the slower frequency.

**Figure 4:**
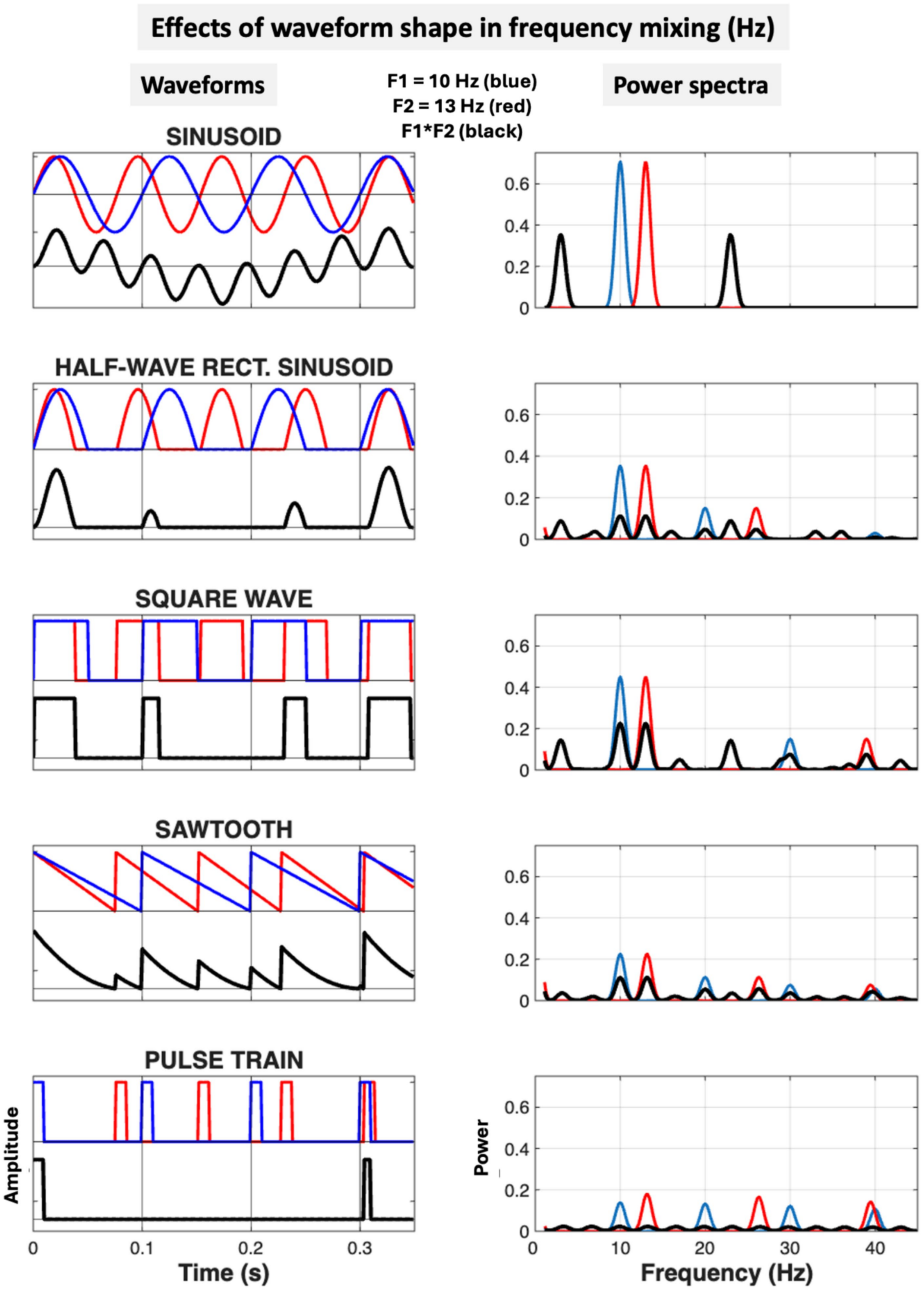
Signal mixing. Multiplications of waveforms of different waveshapes with fundamentals at F1 and F2 produce additional frequencies (F2-F1, F1 + F2). Left plots: primary waveforms F1 (blue) and F2 (red) and their multiplied waveforms (F1*F2, black). Right plots: Spectra of primaries F1 (blue) and F2 (red) and their emergent sideband products (F1*F2 -- > F2-F1, F1 + F2, black). Signals (5 s duration) had maximum amplitudes of unity and were analyzed using the Matlab *pspectrum* function.

**Figure 5:**
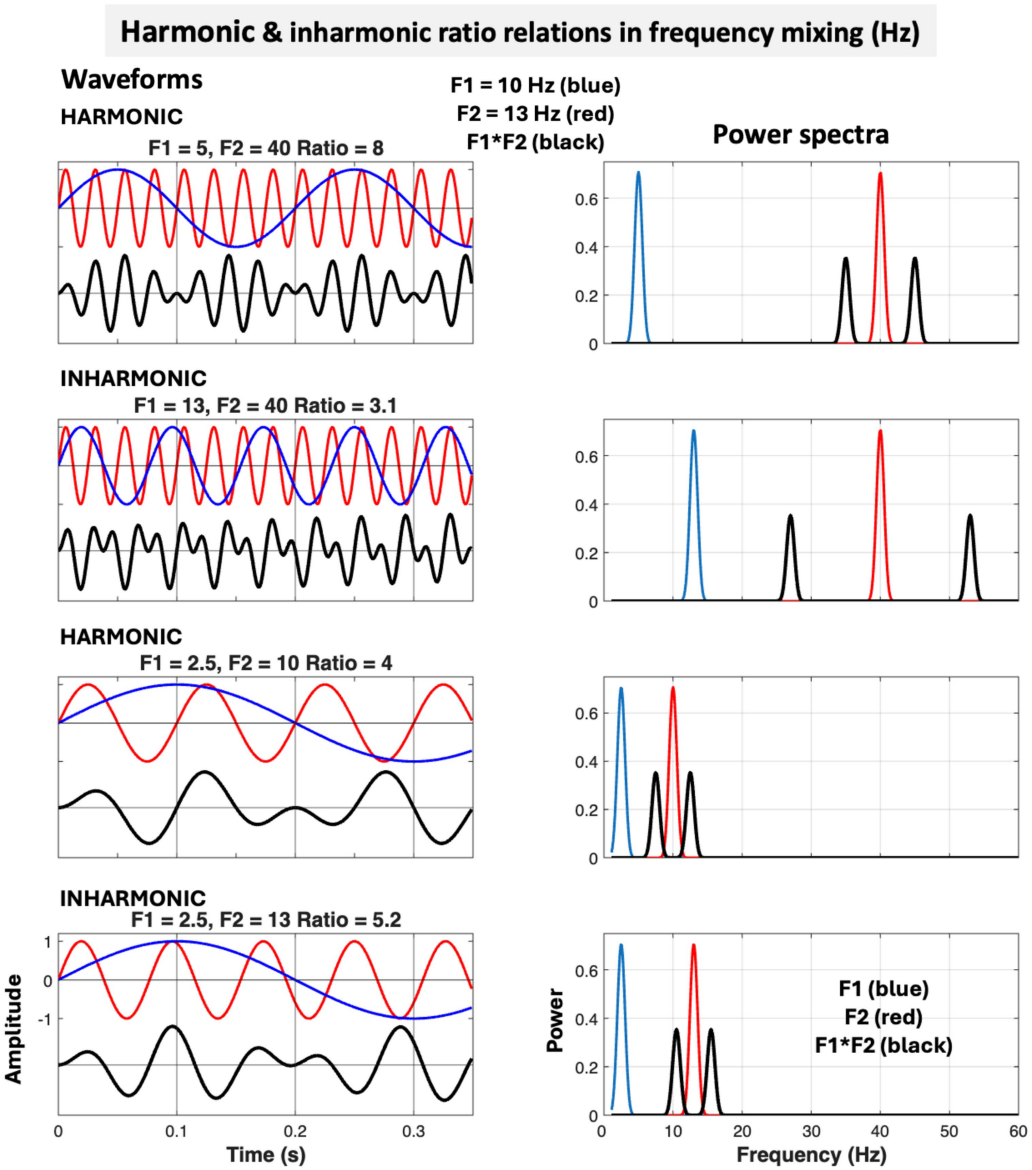
Mixing of waveforms having harmonic and inharmonic frequency ratio relations. Left: primary (F1, F2) and resultant (F1*F2) waveforms for harmonic (integer ratio) and inharmonic (non-integer) ratio relations. The power of the sidebands does not depend on harmonic vs. inharmonic ratio relations.

The cross coupling of these integer-related frequencies is consistent with maximizing the alignment of phase-synchronization between them. The extent of generally recognized brainwave frequency bands (from very high gamma, sharp ripples, to low delta) ranges from roughly 0.5–250 Hz. See Figures 4, 5 for examples of typical neuron mixing and cross frequency couplings.

### 6.3 Entrainment

Entrainment refers to the widely observed synchronization of brainwave oscillations to rhythms of exogenous periodic stimuli (e.g., light, music, etc.), as well as to endogenous rhythms (e.g., heart rate). This process encodes rhythmic stimuli with a temporal phase-locking mechanism. Consequently, neural oscillations are aligned with rhythmic environmental and internal dynamics. The specific mechanisms (e.g., oscillation coupling, stimulus tracking, etc.) supporting entrainment are debated. The presence of multiple concurrent brain oscillations and the role of attention, relative power relationships, etc. further complicate interpretations of entrainment (Lakatos et al., 2008; Lakatos et al., 2019; Duecker et al., 2024).

### 6.4 Coupling of oscillations can alter phase relations and power spectra

Power relations between interacting oscillations typically change as a consequence of linear and nonlinear couplings. When two or more oscillations are linearly synchronized and coupled, phases get reset, and their amplitudes add (constructively and destructively). For oscillations that are excitatory, the coupled oscillations can gain power, enhancing spike generation and the ability to compete with weaker signals. Synchronized inhibitory oscillations can similarly lead to increased boosts in hyperpolarization and discharge suppression.

By the same mechanism, linearly adding the troughs of two waves together (exhibiting constructive interference) will lead to greater membrane polarization (hyperpolarization), thereby suppressing activity. When two interacting waves of the same frequency are 180 degrees out of phase they will cancel each other (destructive interference). Differences in interacting frequencies and relative phases, can generate intermediate magnitudes.

Synchronized oscillations with m:m or m:n integer-related couplings, especially those that are harmonically related, further increase their power, especially when their frequency phases are aligned. Phase coupling two or more oscillations increases power, influence, and competitive strength. With neuron mixing, two oscillations are multiplied to produce two new oscillations with sum and difference frequencies, and the resulting amplitudes of the sideband and carrier components are more complex. The two sidebands divide their combined amplitudes proportionately. Tracking and correlating voltage dynamics generally, and spectral power profiles specifically, could help elucidate signal interactions and information flow, especially in the context of dynamic tasks.

Computing and determining power relationships between oscillatory frequency bands is complicated by the aperiodic 1/f power law wherein amplitude (the square root of power) is inversely proportional to frequency; that is, amplitude increases as frequency decreases, so delta oscillations are ascribed to have greater power than gamma oscillations. Recent research aims to characterize and disambiguate i/f aperiodic activity. Also, as oscillation frequencies decrease, their cycles have longer durations, indicative of larger potentially more powerful neuronal populations (Buzsáki, 2006; Dickey et al., 2022; Gerster et al., 2022).

### 6.5 Measuring phase and amplitude couplings

Multiple methods have been explored by many for finding, measuring, and assessing oscillation phase, and amplitude couplings and coherences, as well as relating these to a variety of brain states and functions (e.g., Tort et al., 2010; Jirsa and Muller, 2013; Aru et al., 2015; Hyafil et al., 2015; Lowet et al., 2016; Safavi et al., 2023; Yeh et al., 2023). It’s neither easy, nor obvious how to do it.

While reporting on phase-phase cross frequency couplings, Belluscio et al. note that difficulties in determining phase can be further confounded with typically asymmetric (non-sinusoidal) waveforms and other issues (Belluscio et al., 2012). They observed three separate gamma bands, two of which couple to different spiking waveform phases of theta, corresponding to maze task activities and to REM sleep. A recent phase autocorrelation methodology (Myrov et al., 2024) aims to assess the temporal stability, of oscillations. This can be used for splitting canonical frequency bands into narrower subbands, as well as profiling distinctive differences between different frequency bands, including their respective burst patterns.

Empirical data in a recent behavioral task-related study, indicates that correct performance is better predicted by coincident oscillatory transients than by co-occurring oscillation peak amplitudes (Siems et al., 2025). Donoghue et al. thoroughly review methodological concerns for studying neural oscillations and the consequences of different methodologies (Donoghue et al., 2022). They make recommendations for accurately assessing spectral peaks/power, aperiodic activity, cross-frequency coupling measures, etc.

### 6.6 Neural signal mixing

Neural signal mixing, a.k.a. “neuron mixing,” goes further than simply synchronizing/coupling different frequencies with each other. Neuron mixing is a separate nonlinear (multiplicative) process by which two different root frequencies are integrated or “mixed,” to create two *new* sum and difference frequencies, effectively abolishing the original (root) frequencies in the process.

This process by which new frequencies are generated and old frequencies are abolished with multiplicative frequency mixing is determined by standard signal processing principles. Figures 4, 5 illustrate effects of the signal mixing process. Figure 4 demonstrates that the sidebands are generated irrespective of waveform shapes. This is important because neural oscillations are typically non-sinusoidal. Figure 5 shows that emergent sidebands are produced irrespective of whether the frequencies of the two signals being mixed have small integer relations (harmonically related).

#### 6.6.1 Existing neurophysiological evidence for neuronal mixing

Ahrens observed this phenomenon when the intrinsic cortical oscillations of anesthetized rats had their vibrissae periodically stimulated over a range of different frequencies (Ahrens et al., 2002). Some research reports of oscillations of cross-frequency couplings, synchronizations, etc. also reflect likely neuron mixing where three oscillations are observed in an F1, F2, and F2-F1 relationship (e.g., Palva et al., 2005; Haufler and Pare, 2019).

Using both brain slices and *in vivo* single cell recordings, Luff et al. demonstrated precise neuron mixing (generating F2-F1 difference frequencies = 10 Hz) in subthreshold membrane potential oscillations for mice over 3 orders of magnitudes of oscillation frequencies (47 Hz, 57 Hz; 497 Hz, 507 Hz; 4,997 Hz, 5,007 Hz). They demonstrated neuron mixing with both exogenous and endogenous oscillations, as well as effects with pharmacological blocking agents. They demonstrated neuron mixing (with oscillation mixings up to 45 Hz) using EEG recordings in the human brain (Luff et al., 2024) across the five canonical frequency bands (gamma, beta, alpha, theta, delta). Additionally, they observed and measured typical mixing frequency clusters within and across different brain regions. They note that biological signal mixing efficiently facilitates “advanced computational operations,” previously “only seen in modern telecommunications.” These empirical results provide additional support for employing established signal processing and communications principles for helping to understand basic neural mechanisms.

### 6.7 Temporal and spatiotemporal pattern processing

This theory also suggests that an additional mechanism ought to be seriously considered as a basic neural processing principle; namely, Spatiotemporal Pattern Processing (STPP). A variety of distinctive waveforms and associated spiking patterns have long been observed in different brain areas (e.g., Cole and Voytek, 2017; Moore et al., 2017; Das et al., 2024). Temporal spike codes arising from interspike intervals, coincidence detection, delay line effects, correlations, and other characteristics, generate patterns (e.g., memory traces) which are encoded and subsequently reactivated/recalled when similar signals, or portions thereof, are subsequently detected. These temporal signatures can be highly idiosyncratic. They are likely coded and represented by cell populations/ensembles that can be sequentially activated and coupled by spatiotemporal traveling waves carrying similar patterns.

We suggest that patterns can be created, multimodally multiplexed, synchronized, coupled/integrated and mixed with each other to progressively incorporate more associated attributes and context as they are processed. These integrated patterns thereby develop increased specificity, selectivity, and connectivity. This progression of pattern integration expands and integrates the cell assemblies and networks with which they associated, on the basis of signal content. Patterns containing common attributes and associations are thereby flexibly grouped, adapted, integrated, and circulated together in multiple associated brain networks.

Through pattern associations, selectivity, and activations, associative networks can multiplex, grow, and adapt. Cell assemblies of high-level feature/object detector arise through combinations of neural assemblies sensitive to specific associated attributes. The activation of such high level detectors also produce spikes and spike patterns. Examples include selective face perception areas (Kanwisher et al., 1997), speech specific neurons (Chan et al., 2014), etc.

Through STDP mediated by attention and salience, new patterns can be learned, modified, coupled, and reinforced to associate arbitrary features, objects, and events at progressively higher stages. Well-reinforced learned patterns become “chunks” that are rapidly accessed directly without going through lower/intermediate processing stages. These enable us to detect, predict, and efficiently execute habitual sensory, motor, and cognitive operations; e.g., recognize speech, execute practiced golf swings or dance moves, drive accustomed routes, etc.

Neural evidence supporting direct rapid access pathways consistent with chunking, has been recently observed by (Hullett et al., 2025). Using ECoG recordings of human volunteers passively listening to natural speech, they successfully found long-range short-latency parallel connections from sensory nuclei to apical regions in the frontal lobe. Their findings demonstrate a fast parallel path alternative to a much slower step-by-step procedure requiring a serial progression through multiple stages (acoustic phonetics, phones, syllables, words, sentences, concepts) in the canonical cortical speech language hierarchy. Parallel paths for robust information processing enable fast processing when signals are highly predictable, and slower component analysis when signals are more ambiguous. Extending this research further, and its analogs into other hierarchical systems (vision, motor, cognitive) and their integrations with each other, promise to prove instructive in understanding the brain and its mechanisms.

In automatic speech recognition systems, very early acoustic cues (e.g., initial word phone/phone clusters) are often sufficient for rapidly triggering correct full word recognition when the joint probabilities of the system, predicated from all its information sources (acoustics, semantics, syntactics, language, speaker characteristics, etc.) are highly predictive of the correct word or phrase. This kind of rapid high confidence/discriminative analysis reflects a kind of efficient chunking, and is referred to as “rapid match” or “fast match” and has long been productively incorporated into automatic speech recognition systems (Gillick and Roth, 1990). To avoid disconcerting speakers (and avoid a false impression of mind reading), automatic speech recognition systems routinely delay reporting back their results until they detect that a speaker has finished speaking the word(s) being recognized. Current generative AI systems broadly rely on this same kind of predictive power, usually furnished by Large Language Model (LLMs) for their automatic text, speech, image, and coding generation and recognition.

### 6.8 STDP and traveling waves/oscillations

Spike-Timing Dependent Plasticity (STDP) concerns changes in strength (positive and negative) at biological synaptic connections determined by correlations between pre-and post -synaptic spike timing patterns. Coincident spikes produce synaptic potentiation, whereas uncorrelated spike produce synaptic depression. These are instrumental for learning and memory/recall functionalities, as well as synaptic induction and network structure/organization (Song et al., 2000; Rao and Sejnowski, 2001; Feldman, 2012; Gilson et al., 2010; Markram et al., 2011, 2012).

A computational model demonstrating the potential effects of traveling waves on synaptic plasticity has recently been shown to form and preferentially reactivate existing pathways (Orepic et al., 2024; Butler and Cruz, 2025). Reactivation of better reinforced pathways is facilitated and thereby preferred over competing alternatives. The encoding of patterns and the subsequent reactivation of them by associated patterns or components thereof (predictive pattern completion), are critical for memory and other operations. These mechanisms are analogous to the generation and reconstruction of holographic objects. Unique interference patterns (e.g., like holograms) are consequently created, coded, activated, and matched for subsequent reconstruction.

It has been proposed that the phase relationships of oscillation phase coordination could facilitate the encoding of information for efficient learning with STDP by formatting spike-time patterns, i.e., preparing neural networks to selectively respond to different incoming temporal spike patterns. Using simulations, Masquelier et al. have demonstrated that oscillation phase-of-firing coordination with informational spike timing patterns could facilitate efficient STDP learning, as well as subsequent pattern reactivation/recognition, in a single oscillation cycle (Masquelier et al., 2009). The oscillations serve as a means to help format the spike times. The combination of oscillations and coordinated phase-of-firing yields faster STDP learning and decoding speed as contrasted with conventional rate-based codes.

In recent work, Ghadiri et al. developed model simulations to demonstrate that using STDP in combination with network oscillations, neural networks can self-organize to enhance transmission connections by matching oscillation periods/delays (Ghadiri et al., 2025). Consequently, transmission connections with non-matching oscillation periods are suppressed. They suggest that this kind of transmission oscillation delay selectivity may optimize brain network organization for efficient signal transmission and communications.

Across modalities, neural spike patterns, including specific spike burst patterns, characterize informational change; e.g. signal onsets/offsets, object detection/recognition. With increasing interest in examining dynamically changing traveling waves, synchronizations, neural oscillations and oscillatory transients, more attention is being given to tracking and characterizing these patterns, and correlating them with brain functions (Cutsuridis, 2012; Lundqvist et al., 2016; Ede et al., 2018; Balasubramanian et al., 2023; Lundqvist et al., 2024; Boshra et al., 2025; Dugue and Chavane, 2025; Banaie Boroujeni et al., 2026). Ermentrout and Kleinfeld (Ermentrout and Kleinfeld, 2001) have speculated that synchronous activity in the brain prevails in the absence of stimulus-triggered, phase coupling traveling waves (Ermentrout and Kleinfeld, 2001).

## 7 Communications

### 7.1 Cortical communications: signal processing principles

As previously proposed (Cariani and Baker, 2022; Baker and Cariani, 2025), we postulate that the different oscillatory frequency bands may be acting as modulated *carriers* of neurally processed signal information rather than being instantiations of that information themselves. Specific oscillation bands and their interactions, may be associated with the different processing stages and the brain regions through which the primary informational signals are being processed and propagated. If this should empirically prove to be the case, then understanding exactly how these temporal and spectral representations/transforms operate and interact is essential to resolving fundamental neural system mechanisms and functions.

We suggest that neural mixing could provide such a mechanism for signal processing and propagation in the brain. Analogous with established signal processing principles, neural mixing combines two oscillatory frequencies (F1, F2) through a nonlinear, multiplicative operation, to produce two new frequencies. These are the sum frequency (F1 + F2) and the difference frequency (F2-F1). In neural tissue, both sum and difference frequencies can be produced, (Luff et al., 2024), albeit with different amplitudes.

In the process, mixing also abolishes the original oscillation frequencies. The sum and difference frequencies constitute the upper and lower sidebands, respectively. In physiological contexts, as in radio signal processing (see section 7 below), the upper sideband (F1 + F2) can be suppressed. The mixing of frequencies can lead to some vestigial remnants of the original frequencies and harmonics of their products.

**Figure 6:**
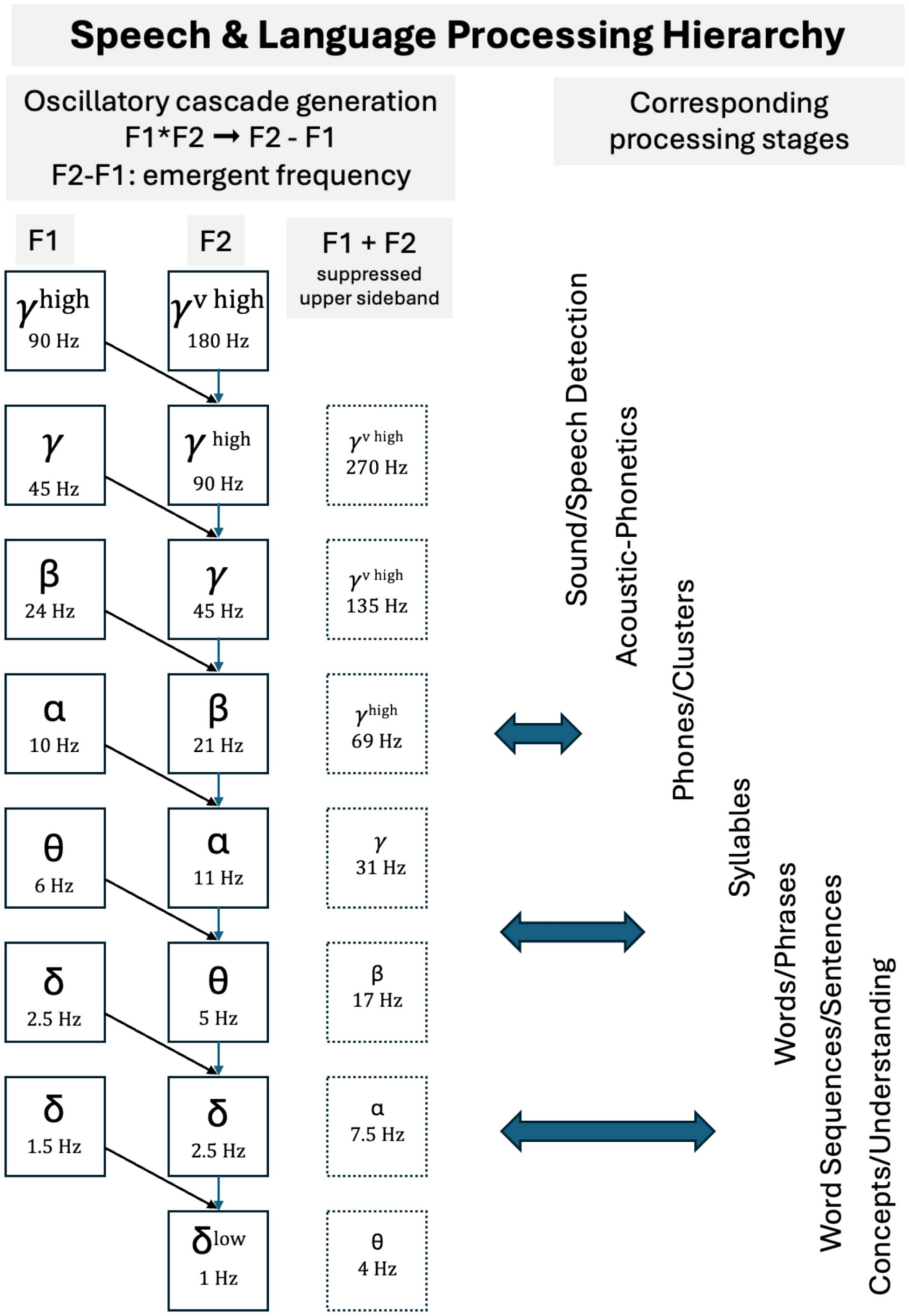
Emergent generation of new frequency bands in an oscillatory cascade. Left boxes: typical center frequencies of commonly observed endogenous oscillation bands. Center boxes: oscillatory frequencies produced by neuronal mixing processes (F1= left box frequency, F2= center box frequency; F2-F1= resultant frequency (lower frequency sideband); Right dotted outline boxes: the suppressed upper frequency sideband F1 + F2. This cascade provides a potential explanation for the proliferation of oscillatory frequencies in brain regions.

Figure 6 shows how a cascade of neural mixing processes can produce a series of emergent oscillations. It shows the primary oscillatory frequencies whose waveform multiplication produces new oscillations at the difference frequencies (F2-F1) and the sum frequencies (F1 + F2). In this cascade mixing model the sum frequencies (upper sidebands) are subsequently attenuated. The degree to which attenuation of these upper sidebands occurs in neural contexts remains to be investigated.

The sidebands (upper and lower) redundantly contain all the actual signal information. Eliminating either sideband and the carrier itself saves considerable power and bandwidth, for improved efficiency and noise robustness. The correspondence of observed human and other animal behaviors which progressively invoke lower oscillation frequencies and their cross-frequency interactions (theta/gamma interactions) as signals progress through higher orders/stages of processing, directly favors using the lower sideband for neural communication.

As illustrated in Figure 6, we suggest this mechanism could be broadly applied across and between the extensive range of canonical frequency bands observed in the brain, and could plausibly provide a means for integrating and communicating information across different brain regions, both locally and globally. Other studies relating neuron mixing in the brain to signal communications, are consistent with this theory (Fujisawa and Buzsaki, 2011; Luff et al., 2024).

The speech and language processing hierarchy of Figure 6 illustrates an example of iterative neuron mixing where two frequencies (F1 and F2) are nonlinearly combined to produce two new sum and difference frequencies (F1 + F2, F2-F1). As previously described, the upper sideband (F1 + F2) and the carrier frequency are attenuated or eliminated with a low pass filter. Consequently, the remaining F2-F1 sideband then becomes the *new* carrier (F2), which can subsequently be mixed with new lower F1 frequencies.

The left column represents a succession of F1 frequencies. The right column represents a succession of F2 carrier frequencies. When each of these F2 frequencies is mixed with the corresponding F1 frequency (see converging arrows), a *new* F2-F1 frequency is generated at the next stage. This succession of mixing stages generates a progression of lower frequencies, that correspond and relate to the canonical frequency bands observed in the brain from gamma to delta.

A hypothetical numerical example is provided to demonstrate this operation (Figure 6). This example illustrates how signal mixing might operate in a hierarchical speech and language processing oscillatory cascade with a 2:1 m:n (high to low frequency sequence) between neighboring frequency bands (see Sections 6.1, 6.2).

For any observed oscillation center point frequency F2, mix it multiplicatively with the next lower frequency band F1, to produce F1 + F2 and F2-F1 frequencies. Take the lesser of these two values (F2-F1) and mix it multiplicatively with the next lower frequency band. Iterate this process, with the F2-F1 products being successively mixed with the next lower frequency band.

Each F1 stage in the left column is designated by an approximate midpoint frequency that is characteristic of that frequency band (e.g., 10 Hz for the 8 Hz -12 Hz alpha band). When that F1 frequency (10 HZ) is mixed with the corresponding F2 beta frequency (21 Hz), the resulting F2-F1 frequency is a new alpha frequency (21–10 = 11 Hz) which becomes the next F2 carrier frequency. At the next mixing stage, an F1 theta of 6 Hz is mixed with the F2 (11 Hz) to generate a new F2-F1 (11–6 Hz) theta at 5 Hz. Despite the rough starting estimates for frequency band midpoints (known to vary as a function of multiple parameters), the series of emergently generated F2-F1 frequency midpoints, closely corresponds to those observed in the canonical gamma to delta frequency bands.

In order to make mixing model predictions regarding the frequencies one should expect to appear in the power spectra of two interacting populations, one would need to make estimates of F1 and F2 when the populations are not interacting, compute the expected power spectra when they are interacting through mixing, and then determine the degrees to which sidebands (F2-F1 and F1 + F2) have appeared. Empirically, power spectra predicted by the signal mixing model and that of observed power spectra of interacting regions could be compared.

In practice, as many neural oscillations are not sinusoidal, characteristic waveshapes could be estimated from observed waveforms by using running autocorrelations to estimate periods, using cross-correlations to phase-align successive periods, and then averaging over multiple aligned cycles. To estimate the relative effects of additive and multiplicative interactions when the populations become coupled, the waveshapes can then be added and/or multiplied (mixed). The resultant waveshapes and power spectra can then be computed and compared with their observed counterparts.

Appearance of sidebands (F2-F1 and F1 + F2) and their relative amplitudes indicates neural mixing. The relative persistence of the primaries (F1, F2) and absence of sidebands are signs of additive interactions. We assume that the waveforms of the two populations (x, y) will have their own dominant independent oscillatory frequencies (F1, F2) when they are not interacting. That interaction may result from additive and/or multiplicative processes. For each population, their effects can be described by interaction terms, additive (x + y) and multiplicative (x*y). These may have different relative weights w_add_ and w_mix_. The sum of these weighted contributions, (w_add_ (x + y) + w_mix_(x*y)) constitutes the predicted interaction waveform. The relative weights that best predict observed waveforms (and hence power spectra) during interaction then need to be fitted. These analyses thus can provide insights into the relative effects of the two operations and their underlying neural mechanisms.

Also shown in Figure 6 is the rough correspondence of different cortical frequency bands, and the interactions of these, to those commonly observed for humans engaged in different speech and language processing stages. These stages are not totally separable (e.g., syllables are sometimes words, etc.) and often locally overlap. Nonetheless the progression of human speech and language processing stages appears to closely relate to observed corresponding high to low frequency bands. For example, gamma rhythms corresponding to acoustic phonetic processing stages, precede (are nested within) lower frequency theta rhythms corresponding to later syllable/words processing stages. Exact correspondences are debated (Giraud and Poeppel, 2012; Ghitza et al., 2013; Molinaro and Lizarazu, 2018). As previously discussed (Section 3.1), given frequency bands are known to play multiple functional roles. It may well be instructive to explore similar correspondences of frequency band progressions to sequential processing stages in other hierarchical cortical systems, such as image/object recognition.

### 7.2 Radio communication principles: single sidebands

Iterated neuron mixing mechanisms resemble well-established iterated signal processing operations in radio communications theory. Key among these are Single Side Band Suppressed Carrier SSB-SC operations, hereafter referred to as SSB (Koomans, 1939; Crecraft and Gergely, 2002; Nahin, 2024). The mathematics underlying neuron mixing and SSB communications are virtually identical, following standard signal processing principles.

SSB communications are a refinement and improvement of superheterodyne amplitude modulated (AM) radio transmissions. Originally patented by Carson in 1915, a host of SSB applications rapidly evolved, spanning long range radio and telephone communications for marine, aviation, military and civilian use as well as audio filtering and data processing (Carson, 1915; Oswald, 1956; Ma, 2023). Our proposed correspondence notably applies the same communications theory principles to neural systems, despite the significant (orders of magnitude) disparity in frequency ranges between the low neuron mixing frequencies and the much higher frequencies characteristic of standard SSB electronic communications.

AM radio broadcasts and transmits carrier frequencies which are amplitude modulated by informational signals (e.g., speech, music), subsequently detected by an antenna tuned to those carrier frequencies, and demodulated by a receiver to regenerate the original signals transmitted. The modulated transmission initially contains two informationally redundant sidebands (F1 + F2, F2-F1), as well as the host carrier frequency. Single Side Band radio eliminates one sideband. Optionally the carrier frequency is also suppressed, thereby improving efficiency, conserving bandwidth, and focusing power in the preserved single sideband. In the neural radio model below, low pass filtering eliminates both the upper sideband as well as the carrier frequency, to yield only the lower sideband. This automatically generated lower sideband becomes the new carrier frequency for the next stage, which subsequently can be mixed with new signal frequencies.

As we have previously proposed, the iteration of a series of neuron mixing stages can produce a cascade of emergent oscillations comparable to AM radio intermediate frequency mixing stages (Cariani and Baker, 2022; Baker and Cariani, 2025). To illustrate this theory, an example (Figure 4) is drawn from the speech/natural language processing cognitive hierarchy. It further correlates a series of typical empirical observations, associated behaviorally with specific oscillation bands. These correspond to progressive stages of spoken language coding, processing, and decoding which are required for the interpretation and understanding of spoken natural language.

In order to avoid the imprecision inherent in relating broad/coarse oscillation frequency ranges to each other, an illustrative numerical example is presented. An approximate characteristic midpoint frequency is used to represent each frequency band which is mixed with another carrier frequency band. This cascade model demonstrates a plausible sequential correspondence of processing stages across eight canonical oscillation bands, from very high gamma to low delta. In this illustration, *each stage* is emergently generated by the nonlinear mixing of frequencies contributed by other stages. Consequently the *entire series* of speech/language stages is emergently generated by the signals themselves, another example of signal centricity and self-organization.

SSB is a well-established *communications* methodology for efficient, reliable, long distance signal transmission. Radio communications aim to faithfully transmit given signals (e.g., speech, music), largely unchanged from the transmitter to the receiver. In sharp contrast, neural signals and signal patterns are extensively processed/transformed during the transmission process.

So although SSB does not tell you *how* neural signals are being progressively processed during their transmission throughout the cortex, tracking SSB oscillations and their interactions might be a productive tool for accessing *where* and *when* different stages of neural processing are occurring and interacting. Appropriate testing is clearly required to explore these possibilities.

### 7.3 More radio communication analogies

There are a number of analogies that can and have been made between electronic and communication systems and the brain. These start with standard electronic components (e.g., diodes, resistors) and proceed to system components and processing methodologies (e.g., matched filters, product detectors). Provocative ideas suggesting that temporally and/or spectrally assembling and correlating neural patterns that are then communicated by carrier oscillations and subsequently decoded, have previously been proposed; e.g., (Engel et al., 1992). Packet switching comes to mind. Like communication packet switching networks, neural pattern information itself may be distributed as bursts (Lundqvist and Wutz, 2022) and/or packets (Luczak et al., 2015) across multiple cell assemblies and then reassembled.

Another example is looking at neural analogs as lock-in amplifiers that holographically use carrier waves (as reference beams) and low pass filtering to increase SNR, as well as to demodulate and extract weak signals from noisy environments. Recent work by Han et al. with non-human primates report on working memory performance effects of radar-like sweeps of prominent phase-locked theta traveling waves across the cortex, which are coupled to beta (Han et al., 2026). Performance was found to vary with phase of the theta wave at the time of stimulus onset. Strong phase-locked theta oscillations associated with saccades in conjunction with visual search tasks have similarly been observed (Xie et al., 2022). Ito et al. further observed phase-amplitude coupling between slow, delta/theta oscillations and faster, alpha-beta and low gamma oscillations (Ito et al., 2013).

## 8 Empirical testing of neural holography and oscillatory cascades

Theories and models of neural holography and oscillatory cascades are presently in a formative state. Many different neural activity patterns and substrates could conceivably support the kinds of neural interference patterns, representations, and operations that are similar to those of holography. Much more empirical neurophysiological work needs to be done in observing and analyzing oscillatory interactions within and between neural populations. More experimental work also needs to better connect oscillatory patterns with functions and behaviors.

### 8.1 Testing neural holography theories

Testing will involve several stages: (1) finding evidence of neural interference patterns, (2) analyze for correlations between interference patterns and stimuli, actions, internal states, (3) show that the correlated neural activity patterns are causal for functions and behaviors. Experiments could impose, abolish, modify or perturb interference patterns and observe whether the patterns themselves result in function/behavior.

The first step is to look for possible neural bases for reliable, robust interference patterns. Temporal spike codes and traveling waves based on neuronal conduction velocities are attractive in this respect because of their invariant natures. Once such candidate signal interactions were identified, their relations to functions could be investigated. One would want to determine whether such patterns are regenerated during working memory and memory retrieval by comparing neuronal responses in successful recognition trials with erroneous ones. The neural responses in successful trials should shed light on decoding (wavefront reconstruction) processes. Finally, perturbative interventions (e.g., external stimuli or pharmacological agents) that disrupted candidate interference patterns could probe the causal connection between the patterns, information retrieval, and informational functions.

### 8.2 Testing the oscillatory cascade model

Similar empirical testing strategies could be used to test the oscillatory cascade model presented in Section 7, where the interference patterns are additive and multiplicative (mixing)interactions that occur between oscillations. Our theory proposes/predicts that the neuronal mixing processing may play a major role in integrating/coordinating neural networks and efficiently communicating information throughout the brain. We further suggest that neuron mixing may provide a principled mechanism by which canonical frequency bands may be emergently generated and abolished. See Communications (Section 7) for more details, descriptions, and expansions on these hypotheses.

How could the specifics of such a model be empirically tested? The first set of questions concerns the existence and extent of coupling based on neural mixing processes *in vivo*. The second set concerns the functional role(s) of such couplings.

First, oscillatory frequencies could be measured under varieties of different conditions: in and across brain regions within and without external visual, auditory, tactile, and electrical stimulation as well as in electrically-driven brain slice preparations. The mixing model predicts that oscillatory sidebands will be produced at specific sum and difference frequencies. The onsets of the couplings of different neural populations typically reset oscillatory phases, so investigators should focus on consistent phase dependencies and emergent oscillatory periodicities, as demonstrated in both brain slices and in vivo (Luff et al., 2024), in time windows immediately following these coupling events. This involves explicitly looking for simultaneous combinations of oscillatory frequencies, e.g., triplets (F1, F2, F2-F1) and quadruplets (F1, F2, F2-F1, F1 + F2), additional harmonics, and common onset phases. One should also observe attenuation/ cessation of F1 and F2 when either F1 + F2 and/or F2-F1 are detected. One should also look for a succession of mixing stages corresponding to progressive (hierarchical) cortical processing stages. It would be very helpful that researchers record and report exact frequency values (Hz) for observed oscillations, and not just broad frequency ranges, e.g., alpha, gamma, etc.

Second, these oscillatory interactions need to be linked directly to perceptual, cognitive, and memory functions and ensuing behavioral performances. Investigators should first determine degrees of correlation (strength of association) of the appearance of emergent oscillatory frequencies with these functions and behaviors. Once correlations are established, then the oscillations can be perturbed or abolished (by confounding stimuli and/or pharmacological means) to determine causality.

## 9 Conclusion

The theory proposed here presents an integrated set of rationales, testable hypotheses, and supporting evidence, to characterize plausible neural processing methods and mechanisms. Starting with traveling waves and spikes, it progresses through a series of signal-centric coding and principled processing steps, to outline a path for achieving cognition and other high order functions and behaviors.

The central theme running through this perspective is the importance of precision timing to the neural system and the processes (e.g., spike coding, phase synchronization/coupling, traveling wave interactions, pattern completion/prediction, object reconstruction) that depend on it.

Fundamentally, the temporal theory we have previously proposed and extended here shifts signal representations and processing operations toward an analog perspective. Temporal correlations of spikes and spike patterns, mediated by neural delay lines and coincidence detectors efficiently process signals and their interactions.

A novel mechanistic theory is outlined to explain how the brain might work to capture, integrate, and process information, in performing its observed functions and behaviors. It takes a broad multidisciplinary perspective using basic physics, signal processing and communication principles, consistent with empirical neurophysiological, psychophysical, and behavioral evidence. This perspective proposes “connecting the dots” by combining and integrating temporal spike codes (e.g., ISIs), traveling waves, holographic principles (using interference patterns, for pattern (re)construction, completion, prediction), recurrent brain architectures with delay lines, spatiotemporal pattern processing (synchronizations, coupling, and neuron mixing), and efficient signal-centric communications, including those analogous to single sideband communications. These phenomena are deeply integrated with each other in a hybrid neural system that essentially combines analog (continuous) and temporally discrete (e.g., spikes, onsets) operations. The theory proposed here presents a set of rationales, testable hypotheses, and supporting evidence, to characterize neural processing methods and mechanisms.

Temporal precisions and phases matter. Traveling waves can record objects, events, etc. with holographic-like interference patterns of spike generated patterns and sequences. These patterns can subsequently be reactivated for memory retrieval, replay, cell assembly, etc. Traveling waves, can broadly and directionally distribute information, which may travel as packets. Oscillations could operate as carriers of information throughout multiple neural processing stages. Proposed mechanisms apply phase-locking, phase coherence, holographic, and signal processing principles to enable synchronized oscillation band couplings. Neural mixing is proposed for the emergent generation and abolition of some frequency bands.

Temporal precision and phase drive precise synchronization and coupling processes. When oscillations interact, they do so at precise points in time, with phase resets that synchronize, coordinate, and amplify signals. When oscillations interact, synchronize, couple, mix, etc., *new* temporally precise signals may be initiated. Older temporally precise signals may or may not be preserved. The interactions of traveling waves, especially those directionally focused, can selectively reactivate cell assemblies, thereby amplifying, recalling, reconstructing, and integrating existing informational patterns (e.g., memory, chunked motoric patterns, cognitive patterns, etc.).

To summarize, cell populations are created, coordinated, and activated by waves of synchronized electrical activity, spike trains, and the temporal patterns (e.g., interspike intervals) that these generate. Electrical activity, both subthreshold voltage changes and superthreshold spikes, result from the combined excitatory and inhibitory electrical influences to which they are exposed. Phase-locking preserves the timing of spikes, temporal resolution of their patterns, and the information contained in them. These patterns spread by multiple means (synaptically, ephaptically, through gap junctions, electrical fields, etc.). Architecturally, these operations are well-supported by a dense mesh of highly interconnected recurrent networks, largely self-organized and dynamically adaptable through plasticity processes.

Traveling waves spread electrical excitation locally and globally across networks. Populations of associated cells multiplex, synchronize, and couple to create new spatiotemporal patterns. These can respond selectively to pre-existing spatiotemporal patterns (e.g., associated memory traces). These waves broadcast information and distribute it widely through sets of multiple parallel channels (delay lines), characterized by different velocities (e.g., faster myelinated fibers, slower unmyelinated fibers, etc.), synaptic junctions, etc. Multiple traveling waves interact with each other to form interference patterns, facilitating and suppressing local regions with linear constructive and destructive interference. We suggest that traveling waves are instrumental in coding (modulating) and decoding (demodulating) signal information as well as distributing, activating/suppressing, coordinating/organizing, and propagating information through multiple synchronization/coupling stages.

Our theory proposes that temporal and spectral processes are synchronized and coupled at multiple levels on the basis of specific phase coherence alignments and spatiotemporal pattern correlations, as signals are routed and progress through multiple processing stages. Standard nonlinear signal processing methodologies create and abolish oscillation frequencies through cross-frequency neuronal mixing processes. Based on prior work invoking communication principles and observed neural behaviors, we suggest that oscillations are plausibly carriers for propagating neural signal representations. Using single sidebands, we describe how emergently generated oscillatory cascades could carry and integrate information across the brain both locally and globally.

Specific oscillation bands can serve different functions in different contexts and processing stages. Particular oscillation bands may reflect given processing stages and/or functional states (e.g., attentional control). Shifts from one oscillation band to another may reflect a change between neural processing states in the progression of signal processing stages. This theory proposes that both oscillatory and non-oscillatory transients may serve as distinctive markers between neural processing states, within and across different processing stages.

Empirical testing is essential for accepting, rejecting, and modifying these hypotheses. Independent and joint collaborations are welcomed for exploring these ideas and their consequences. To the degree to which this theory and related hypotheses are supported by current and future research, they have the potential to shift the paradigm for better studying and understanding the brain.

## Data Availability

The original contributions presented in the study are included in the article/supplementary material, further inquiries can be directed to the corresponding author/s.

## References

[ref1] Abeles M. (1982a). Local Cortical Circuits. An Electrophysiological Study. Berlin: Springer-Verlag.

[ref2] Abeles M. (1982b). Role of the cortical neuron: integrator or coincidence detector. Isr. J. Med. Sci. 18, 83–92, 6279540

[ref3] Abeles M. (2003). “Synfire Chains,” in The Handbook of Brain Theory and Neural Networks, ed. Arbib M. A.. 2nd ed (Cambridge, MA: MIT Press), 1143–1146.

[ref4] Ahissar E. (1998). Temporal-code to rate-code conversion by neuronal phase-locked loops. Neural Comput. 10, 597–650. doi: 10.1162/089976698300017683, 9527836

[ref5] Ahissar E. Nelinger G. Assa E. Karp O. Saraf-Sinik I. (2023). Thalamocortical loops as temporal demodulators across senses. Commun Biol 6:562. doi: 10.1038/s42003-023-04881-4, 37237075 PMC10220046

[ref6] Ahrens K. F. Levine H. Suhl H. Kleinfeld D. (2002). Spectral mixing of rhythmic neuronal signals in sensory cortex. Proc. Natl. Acad. Sci. U. S. A. 99, 15176–15181. doi: 10.1073/pnas.222547199, 12403828 PMC137563

[ref7] Alamia A. VanRullen R. (2019). Alpha oscillations and traveling waves: signatures of predictive coding? PLoS Biol. 17:e3000487. doi: 10.1371/journal.pbio.3000487, 31581198 PMC6776260

[ref8] Alamia A. VanRullen R. (2024). A traveling waves perspective on temporal binding. J. Cogn. Neurosci. 36, 721–729. doi: 10.1162/jocn_a_02004, 37172133

[ref9002] Almeida-Filho D. G. Lopes-dos-Santos V. Vasconcelos N. A. P. Miranda J. G. V. Tort A. B. L. Ribeiro S. (2014). An investigation of Hebbian phase sequences as assembly graphs. Frontiers in Neural Circuits 8:34. doi: 10.3389/fncir.2014.00034. PMC3986516 24782715

[ref9] Aru J. Aru J. Priesemann V. Wibral M. Lana L. Pipa G. . (2015). Untangling cross-frequency coupling in neuroscience. Curr. Opin. Neurobiol. 31, 51–61. doi: 10.1016/j.conb.2014.08.002, 25212583

[ref10] Baker J. M. (1975). A New Time-Domain Analysis of Human Speech and Other Complex Waveforms (Ph.D. thesis, Computer Science). Pittsburgh: Carnegie Mellon University.

[ref11] Baker J. M. (1979). *Performance Statistics of the HEAR Acoustic Processor Proceedings IEEE ICASSP*. Washington, D.C, pp. 262–265.

[ref12] Baker J. M. Baker J. K. Lettvin J. Y. (1972). More visible speech. J. Acoust. Soc. Am. 52:183. doi: 10.1121/1.1982148

[ref13] Baker J. M. Cariani P. (2025). Time-domain brain: temporal mechanisms for brain functions using time-delay nets, holographic processes, radio communications, and emergent oscillatory sequences. Front. Comput. Neurosci. 19:1540532. doi: 10.3389/fncom.2025.1540532, 40041740 PMC11877394

[ref14] Balasubramanian K. Arce-McShane F. I. Dekleva B. M. Collinger J. L. Hatsopoulos N. G. (2023). Propagating motor cortical patterns of excitability are ubiquitous across human and non-human primate movement initiation. iScience 26:106518. doi: 10.1016/j.isci.2023.106518, 37070071 PMC10105290

[ref15] Banaie Boroujeni K. Helfrich R. F. Fiebelkorn I. C. Bentley J. N. Brunner P. Lin J. J. . (2026). High-frequency bursts facilitate fast communication for human spatial attention. Nat. Neurosci. 29, 435–444. doi: 10.1038/s41593-025-02160-5, 41331141 PMC12695012

[ref16] Barsellino A. Poggio T. (1972). Holographic aspects of temporal memory and optomotor responses. Kybernetik 10, 58–60. doi: 10.1007/BF00288785, 4338085

[ref17] Belluscio M. A. Mizuseki K. Schmidt R. Kempter R. Buzsáki G. (2012). Cross-frequency phase-phase coupling between theta and gamma oscillations in the hippocampus. J. Neurosci. 32, 423–435. doi: 10.1523/JNEUROSCI.4122-11.2012, 22238079 PMC3293373

[ref18] Benigno G. B. Budzinski R. C. Davis Z. W. Reynolds J. H. Muller L. (2023). Waves traveling over a map of visual space can ignite short-term predictions of sensory input. Nat. Commun. 14:3409. doi: 10.1038/s41467-023-39076-2, 37296131 PMC10256723

[ref19] Bennett W. R. (1953). A machine for continuous display of short-term correlation – “the correlatograph”. Bell Syst. Tech. J. 32, 1173–1185.

[ref20] Berger H. (1929). Uber das Elektrenkephalogramm des Menschen. Arch. Psychiatr. Nervenkr. 87, 527–570. doi: 10.1007/BF01797193

[ref21] Beurle R. L. (1956). Properties of a mass of cells capable of regenerating pulses. Philos. Trans. R. Soc. Lond. B240, 55–94.

[ref22] Bhattacharya S. Brincat S. L. Lundqvist M. Miller E. K. (2022). Traveling waves in the prefrontal cortex during working memory. PLoS Comput. Biol. 18:e1009827. doi: 10.1371/journal.pcbi.1009827, 35089915 PMC8827486

[ref23] Biddulph R. (1954). Short-term autocorrelation analysis and correlatograms of spoken digits. J. Acoust. Soc. Am. 26, 539–541.

[ref24] Biel A. L. Minarik T. Sauseng P. (2021). EEG cross-frequency phase synchronization as an index of memory matching in visual search. NeuroImage 235:117971. doi: 10.1016/j.neuroimage.2021.117971, 33839263

[ref25] Boshra R. Harris M. Dougherty K. Berg M. Morea B. M. Alitto H. J. . (2025). Causal role for pulvinar Burst firing in Thalamo-cortical Attention Control. bioRxiv 2025:676591. doi: 10.1101/2025.09.16.676591

[ref26] Braitenberg V. Heck D. Sultan F. (1997). The detection and generation of sequences as a key to cerebellar function. Experiments and theory. Brain Behav. Sci. 20, 229–277, 10096998

[ref27] Breakspear M. (2017). Dynamic models of large-scale brain activity. Nat. Neurosci. 20, 340–352. doi: 10.1038/nn.4497, 28230845

[ref28] Brindley G. S. (1964). The use made by the cerebellum of the information that it receives from sense organs. Int. Brain Res. Program. Bull. 3:80.

[ref29] Brindley G. S. (1969). Nerve net models of plausible size that perform many simple learning tasks. Proc. R. Soc. Lond. B Biol. Sci. 174, 173–191. doi: 10.1098/rspb.1969.0087, 4391179

[ref30] Brody C. D. Hopfield J. J. (2003). Simple networks for spike-timing-based computation, with application to olfactory processing. Neuron 37, 843–852. doi: 10.1016/s0896-6273(03)00120-x, 12628174

[ref31] Bryngdahl O. Lohmann A. (1968). Single-sideband holography. J. Opt. Soc. Am. 58, 620–624.

[ref32] Buracas G. T. Zador A. M. DeWeese M. R. Albright T. D. (1998). Efficient discrimination of temporal patterns by motion-sensitive neurons in primate visual cortex. Neuron 20, 959–969. doi: 10.1016/S0896-6273(00)80477-8, 9620700

[ref33] Busch A. Roussy M. Luna R. Leavitt M. L. Mofrad M. H. Gulli R. A. . (2024). Neuronal activation sequences in lateral prefrontal cortex encode visuospatial working memory during virtual navigation. Nat. Commun. 15:4471. doi: 10.1038/s41467-024-48664-9, 38796480 PMC11127969

[ref34] Butler K. Cruz L. (2025). Neuronal traveling waves form preferred pathways using synaptic plasticity. J. Comput. Neurosci. 53, 181–198. doi: 10.1007/s10827-024-00890-2, 39729255 PMC11868204

[ref35] Buzsáki G. (2006). Rhythms of the Brain. Oxford: Oxford University Press.

[ref36] Canolty R. T. Edwards E. Dalal S. S. Soltani M. Nagarajan S. S. Kirsch H. E. . (2006). High gamma power is phase-locked to theta oscillations in human neocortex. Science 313, 1626–1628. doi: 10.1126/science.1128115, 16973878 PMC2628289

[ref37] Canolty R. T. Knight R. T. (2010). The functional role of cross-frequency coupling. Trends Cogn. Sci. 14, 506–515. doi: 10.1016/j.tics.2010.09.001, 20932795 PMC3359652

[ref38] Cariani P. (1997). Emergence of new signal-primitives in neural networks. Intellectica 1, 95–143.

[ref39] Cariani P. Baker J. M. (2022). Time is of the essence: neural codes, synchronies, oscillations, architectures. Front. Comput. Neurosci. 16:898829. doi: 10.3389/fncom.2022.898829, 35814343 PMC9262106

[ref40] Cariani P. Baker J. M. (2025). Survey of temporal coding of sensory information. Front. Comput. Neurosci. 19:1571109. doi: 10.3389/fncom.2025.1571109 40672999 PMC12263675

[ref41] Cariani P. Baker J. M. (2026). Temporal codes and recurrent timing nets for rhythmic expectancy. Front. Comput. Neurosci. 20:1814579. doi: 10.3389/fncom.2026.1814579 42221575 PMC13219049

[ref42] Cariani P. Delgutte B. (1996). Neural correlates of the pitch of complex tones. I. Pitch and pitch salience. II. Pitch shift, pitch ambiguity, phase-invariance, pitch circularity, and the dominance region for pitch. J. Neurophysiol. 76, 1698–1734. doi: 10.1152/jn.1996.76.3.1717, 8890287

[ref43] Carr C. E. (1993). Processing of temporal information in the brain. Annu. Rev. Neurosci. 16, 223–243. doi: 10.1146/annurev.ne.16.030193.001255, 8460892

[ref44] Carrillo-Reid L. Han S. Yang W. Akrouh A. Yuste R. (2019). Controlling visually guided behavior by holographic recalling of cortical ensembles. Cell 178, 447–457.e5. doi: 10.1016/j.cell.2019.05.045, 31257030 PMC6747687

[ref45] Carson J. R. (1915). *Circuits using the same Frequency for two Directions of Communication*.

[ref46] Cavanaugh J. P. (1975). “Two Classes of Holographic Processes Realizable in the Neural Realm,” in Formal Aspects of Cognitive Processes. Lecture Notes in Computer Science, eds. Storer T. Winter D. (Berlin: Springer).

[ref47] Chan A. M. Dykstra A. R. Jayaram V. Leonard M. K. Travis K. E. Gygi B. . (2014). Speech-specific tuning of neurons in human superior temporal gyrus. Cereb. Cortex 24, 2679–2693. doi: 10.1093/cercor/bht127, 23680841 PMC4162511

[ref48] Chen B. Ciria L. F. Hu C. Ivanov P. C. (2022). Ensemble of coupling forms and networks among brain rhythms as function of states and cognition. Commun Biol 5:82. doi: 10.1038/s42003-022-03017-4, 35064204 PMC8782865

[ref49] Chopping P. T. (1968). Holographic model of temporal recall. Nature 217, 781–782. doi: 10.1038/217781a0, 4295942

[ref50] Chung S. H. Lettvin J. Y. Raymond S. A. (1974). Proceedings: the CLOOGE: a simple device for interspike interval analysis. J. Physiol. 239, 63–66, 4547285 PMC1330913

[ref51] Chung S. H. Raymond S. A. Lettvin J. Y. (1970). Multiple meaning in single visual units. Brain Behav. Evol. 3, 72–101. doi: 10.1159/000125464, 5522353

[ref52] Cole S. R. Voytek B. (2017). Brain oscillations and the importance of waveform shape. Trends Cogn. Sci. 21, 137–149. doi: 10.1016/j.tics.2016.12.008, 28063662

[ref53] Crecraft D. I. Gergely S. (2002). “9 - Radio Communication Techniques,” in Analog Electronics, ed. Crecraft D. I. (Oxford: Butterworth-Heinemann), 200–232.

[ref54] Cruddas J. Pang J. C. Fornito A. (2026). Cortical traveling waves in time and space: physics, physiology, and psychology. Neuron 114, 985–1005. doi: 10.1016/j.neuron.2025.12.019, 41666935

[ref55] Cutsuridis V. (2012). Bursts shape the NMDA-R mediated spike timing dependent plasticity curve: role of burst interspike interval and GABAergic inhibition. Cogn. Neurodyn. 6, 421–441. doi: 10.1007/s11571-012-9205-1, 24082963 PMC3438326

[ref56] Das A. Zabeh E. Ermentrout B. Jacobs J. (2024). Planar, spiral, and concentric Traveling Waves Distinguish Cognitive states in human memory. bioRxiv 2024:577456. doi: 10.1101/2024.01.26.577456 PMC13250086 41963323

[ref57] Das A. Zhang J. Zabeh E. Ermentrout B. Jacobs J. (2018). Hidden Spirals reveal Neurocomputational Mechanisms of Traveling Waves in human memory. bioRxiv 2018:86225. doi: 10.1101/2025.11.03.686225

[ref58] Davis Z. W. Muller L. Martinez-Trujillo J. Sejnowski T. Reynolds J. H. (2020). Spontaneous travelling cortical waves gate perception in behaving primates. Nature 587, 432–436. doi: 10.1038/s41586-020-2802-y, 33029013 PMC7677221

[ref59] Deco G. Jirsa V. K. Robinson P. A. Breakspear M. Friston K. (2008). The dynamic brain: from spiking neurons to neural masses and cortical fields. PLoS Comput. Biol. 4:e1000092. doi: 10.1371/journal.pcbi.1000092, 18769680 PMC2519166

[ref60] Dickey C. W. Verzhbinsky I. A. Jiang X. Rosen B. Q. Kajfez S. Stedelin B. . (2022). Widespread ripples synchronize human cortical activity during sleep, waking, and memory recall. Proc. Natl. Acad. Sci. USA 119:e2107797119. doi: 10.1073/pnas.2107797119, 35867767 PMC9282280

[ref61] Donoghue T. Schaworonkow N. Voytek B. (2022). Methodological considerations for studying neural oscillations. Eur. J. Neurosci. 55, 3502–3527. doi: 10.1111/ejn.15361, 34268825 PMC8761223

[ref62] Duecker K. Doelling K. B. Breska A. Coffey E. B. J. Sivarao D. V. Zoefel B. (2024). Challenges and approaches in the study of neural entrainment. J. Neurosci. 44:e1234242024. doi: 10.1523/JNEUROSCI.1234-24.2024, 39358026 PMC11450538

[ref63] Dugue L. Chavane F. (2025). Traveling waves across scales: different mechanisms but same canonical computation? eLife 14:753. doi: 10.7554/eLife.106753, 41258882 PMC12629594

[ref64] Eccles J. C. (1958). The physiology of imagination. Sci. Am. 199, 135–142. doi: 10.1038/scientificamerican0958-135 13580278

[ref65] Ede F.v. Quinn A. J. Woolrich M. W. Nobre A. C. (2018). Neural oscillations: sustained rhythms or transient burst-events? Trends Neurosci. 41, 415–417. doi: 10.1016/j.tins.2018.04.004, 29739627 PMC6024376

[ref66] Engel A. K. Konig P. Kreiter A. K. Schillen T. B. Singer W. (1992). Temporal coding in the visual cortex: new vistas on integration in the nervous system. Trends Neurosci. 15, 218–226. doi: 10.1016/0166-2236(92)90039-b, 1378666

[ref67] Ermentrout G. B. Kleinfeld D. (2001). Traveling electrical waves in cortex: insights from phase dynamics and speculation on a computational role. Neuron 29, 33–44. doi: 10.1016/s0896-6273(01)00178-7, 11182079

[ref68] Feingold J. Gibson D. J. DePasquale B. Graybiel A. M. (2015). Bursts of beta oscillation differentiate postperformance activity in the striatum and motor cortex of monkeys performing movement tasks. Proc. Natl. Acad. Sci. USA 112, 13687–13692. doi: 10.1073/pnas.1517629112, 26460033 PMC4640760

[ref69] Feldman D. E. (2012). The spike-timing dependence of plasticity. Neuron 75, 556–571. doi: 10.1016/j.neuron.2012.08.001, 22920249 PMC3431193

[ref70] Fell J. Axmacher N. (2011). The role of phase synchronization in memory processes. Nat. Rev. Neurosci. 12, 105–118. doi: 10.1038/nrn2979, 21248789

[ref71] Friedman-Hill S. Maldonado P. E. Gray C. M. (2000). Dynamics of striate cortical activity in the alert macaque: I. Incidence and stimulus-dependence of gamma-band neuronal oscillations. Cereb. Cortex 10, 1105–1116. doi: 10.1093/cercor/10.11.1105, 11053231

[ref72] Fries P. (2015). Rhythms for cognition: communication through coherence. Neuron 88, 220–235. doi: 10.1016/j.neuron.2015.09.034, 26447583 PMC4605134

[ref73] Fujisawa S. Buzsaki G. (2011). A 4 Hz oscillation adaptively synchronizes prefrontal, VTA, and hippocampal activities. Neuron 72, 153–165. doi: 10.1016/j.neuron.2011.08.018, 21982376 PMC3235795

[ref74] Gabor D. (1948). A new microscopic principle. Nature 161:777. doi: 10.1038/161777a0, 18860291

[ref75] Gabor D. (1968). Holographic model of temporal recall. Nature 217, 584–584 (Comment on holographic model of Longuet–Higgins). doi: 10.1038/217584a0, 5641120

[ref76] Gabor D. (1969). Associative holographic memories. IBM J. Res. Dev. 13, 156–159. doi: 10.1147/rd.132.0156

[ref77] Gautrais J. Thorpe S. (1998). Rate coding versus temporal order coding: a theoretical approach. Biosystems 48, 57–65. doi: 10.1016/s0303-2647(98)00050-1, 9886632

[ref78] Gerster M. Waterstraat G. Litvak V. Lehnertz K. Schnitzler A. Florin E. . (2022). Separating neural oscillations from aperiodic 1/f activity: challenges and recommendations. Neuroinformatics 20, 991–1012. doi: 10.1007/s12021-022-09581-8, 35389160 PMC9588478

[ref79] Ghadiri A. Rezaei H. Aertsen A. Kumar A. Valizadeh A. (2025). Delay selection by spike-timing-dependent plasticity shapes efficient networks for signal transmission. bioRxiv 2025:682868. doi: 10.1101/2025.10.20.682868 PMC13470781 42448571

[ref80] Ghazizadeh E. Ching S. (2021). Slow manifolds within network dynamics encode working memory efficiently and robustly. PLoS Comput. Biol. 17:e1009366. doi: 10.1371/journal.pcbi.1009366, 34525089 PMC8475983

[ref81] Ghitza O. Giraud A. L. Poeppel D. (2013). Neuronal oscillations and speech perception: critical-band temporal envelopes are the essence. Front. Hum. Neurosci. 6:340. doi: 10.3389/fnhum.2012.00340, 23316150 PMC3539830

[ref82] Gillick L. S. Roth R. (1990). *Fast Match Technique Proceedings DARPA Speech and Natural Language HLT Conference*. Hidden Valley, PA.

[ref83] Gilson M. Burkitt A. Van Hemmen L. J. (2010). STDP in recurrent neuronal networks. Front. Comput. Neurosci. 4:23. doi: 10.3389/fncom.2010.00023 20890448 PMC2947928

[ref84] Giraud A. L. Poeppel D. (2012). Cortical oscillations and speech processing: emerging computational principles and operations. Nat. Neurosci. 15, 511–517. doi: 10.1038/nn.3063, 22426255 PMC4461038

[ref9001] Gonzalez J. Cavelli M. Mondino A. Rubido N. Bl Tort A. Torterolo P. (2020). Communication through coherence by means of cross-frequency coupling. Neuroscience 449, 157–164. doi: 10.1016/j.neuroscience.2020.09.019. 32926953

[ref85] Goyal A. Miller J. Qasim S. E. Watrous A. J. Zhang H. Stein J. M. . (2020). Functionally distinct high and low theta oscillations in the human hippocampus. Nat. Commun. 11:2469. doi: 10.1038/s41467-020-15670-6, 32424312 PMC7235253

[ref86] Han H. B. Brincat S. L. Buschman T. J. Miller E. K. (2026). Working memory readout varies with frontal theta rhythms. Neuron 114, 159–166.e2. doi: 10.1016/j.neuron.2025.09.031, 41118756

[ref87] Haque H. Wang S. H. Siebenhuhner F. Robertson E. M. Palva J. M. Palva S. (2025). Top-down selection of visual working memory contents is supported by alpha-band phase-synchronized oscillatory networks. Imaging Neurosci (Camb) 3:1034. doi: 10.1162/IMAG.a.1034, 41445561 PMC12723408

[ref88] Haufler D. Pare D. (2019). Detection of multiway gamma coordination reveals how frequency mixing shapes neural dynamics. Neuron 101, 603–614.e6. doi: 10.1016/j.neuron.2018.12.028, 30679018 PMC6384143

[ref89] Hebb D. O. (1949). The Organization of Behavior. New York: Simon and Schuster.

[ref90] Heil P. Irvine D. R. (1997). First-spike timing of auditory-nerve fibers and comparison with auditory cortex. J. Neurophysiol. 78, 2438–2454. doi: 10.1152/jn.1997.78.5.2438, 9356395

[ref91] Heinz G. (2004). *Interference Networks - a Physical, Structural and Behavioural Approach to Nerve System*. In: Brain Inspired Cognitive Systems - BICS2004).

[ref92] Herman P. A. Lundqvist M. Lansner A. (2013). Nested theta to gamma oscillations and precise spatiotemporal firing during memory retrieval in a simulated attractor network. Brain Res. 1536, 68–87. doi: 10.1016/j.brainres.2013.08.002, 23939226

[ref93] Hinton G. E. Anderson J. A. (1989). Parallel Models of Associative Memory. Hillsdale, NJ: Lawrence Erlbaum Associates.

[ref94] Hopfield J. J. (1995). Pattern recognition computation using action potential timing for stimulus representation. Nature 376, 33–36. doi: 10.1038/376033a0, 7596429

[ref95] Hullett P. W. Leonard M. K. Gorno-Tempini M. L. Mandelli M. L. Chang E. F. (2025). Parallel encoding of speech in human frontal and temporal lobes. Nat. Commun. 17:814. doi: 10.1038/s41467-025-67517-7 41461641 PMC12824166

[ref96] Hyafil A. Giraud A. L. Fontolan L. Gutkin B. (2015). Neural cross-frequency coupling: connecting architectures, mechanisms, and functions. Trends Neurosci. 38, 725–740. doi: 10.1016/j.tins.2015.09.001, 26549886

[ref97] Ishizuka K. (1994). Resolution improvement by titled single-sideband holography: preliminary experiments. Ultramicroscopy 53, 9–14. doi: 10.1016/0304-3991(94)90099-X

[ref98] Ito J. Maldonado P. Grun S. (2013). Cross-frequency interaction of the eye-movement related LFP signals in V1 of freely viewing monkeys. Front. Syst. Neurosci. 7:1. doi: 10.3389/fnsys.2013.00001, 23420631 PMC3572441

[ref99] Jacobs M. Budzinski R. C. Muller L. Ba D. Keller T. A. (2025). Preprint. Traveling Waves Integrate Spatial Information through time. arXiv 2025:6034. doi: 10.48550/arXiv.2502.06034

[ref100] Jirsa V. Muller V. (2013). Cross-frequency coupling in real and virtual brain networks. Front. Comput. Neurosci. 7:78. doi: 10.3389/fncom.2013.00078, 23840188 PMC3699761

[ref101] Johansson G. (1973). Visual perception of biological motion and a model for its analysis. Percept. Psychophys. 14, 201–211. doi: 10.3758/BF03212378

[ref102] John E. R. (1972). Switchboard vs. statistical theories of learning and memory. Science 177, 850–864. doi: 10.1126/science.177.4052.850, 5054641

[ref103] Kanwisher N. McDermott J. Chun M. M. (1997). The fusiform face area: a module in human extrastriate cortex specialized for face perception. J. Neurosci. 17, 4302–4311. doi: 10.1523/JNEUROSCI.17-11-04302.1997, 9151747 PMC6573547

[ref104] Kohonen T. (1987). “Content-Addressable Memories,” in Springer Series in Information Sciences, ed. Kohonen T.. 1st ed (Berlin, Heidelberg: Springer-Verlag).

[ref105] Koomans N. (1939). Asymmetric-sideband broadcasting. Proc. IRE 27, 687–690. doi: 10.1109/JRPROC.1939.228803

[ref106] Kopell N. Kramer M. A. Malerba P. Whittington M. A. (2010). Are different rhythms good for different functions? Front. Hum. Neurosci. 4:187. doi: 10.3389/fnhum.2010.00187, 21103019 PMC2987659

[ref107] Kramer M. A. (2022). Golden rhythms as a theoretical framework for cross-frequency organization. Neuron Behav Data Anal Theory 1:38960. doi: 10.51628/51001c.38960 PMC10181851 37186520

[ref108] Kuzmina E. Kriukov D. Lebedev M. (2024). Neuronal travelling waves explain rotational dynamics in experimental datasets and modelling. Sci. Rep. 14:3566. doi: 10.1038/s41598-024-53907-2, 38347042 PMC10861525

[ref109] Lakatos P. Gross J. Thut G. (2019). A new unifying account of the roles of neuronal entrainment. Curr. Biol. 29, R890–R905. doi: 10.1016/j.cub.2019.07.075, 31550478 PMC6769420

[ref110] Lakatos P. Karmos G. Mehta A. D. Ulbert I. Schroeder C. E. (2008). Entrainment of neuronal oscillations as a mechanism of attentional selection. Science 320, 110–113. doi: 10.1126/science.1154735, 18388295

[ref111] Langdon C. Genkin M. Engel T. A. (2023). A unifying perspective on neural manifolds and circuits for cognition. Nat. Rev. Neurosci. 24, 363–377. doi: 10.1038/s41583-023-00693-x, 37055616 PMC11058347

[ref112] Lange F. H. (1967). Correlation Techniques. Princeton: Van Nostrand.

[ref113] Lashley K. S. (1929). Brain Mechanisms and Intelligence; A Quantitative Study of Injuries to the Brain. Chicago, Ill: The University of Chicago Press.

[ref114] Leith E. N. Upatnieks J. (1962). Reconstructed wavefronts and communication theory. J. Opt. Soc. Am. 52, 1123–1130. doi: 10.1364/josa.52.001123

[ref115] Liboni L. H. B. Budzinski R. C. Busch A. N. Löwe S. Keller T. A. Welling M. . (2025). Image segmentation with traveling waves in an exactly solvable recurrent neural network. Proc. Natl. Acad. Sci. 122:e2321319121. doi: 10.1073/pnas.2321319121, 39752524 PMC11725882

[ref116] Licklider J. C. R. Pollack I. (1948). Effects of differentiation, integration, and infinite peak clipping upon the intelligibility of speech. J. Acoust. Soc. Am. 20, 42–51. doi: 10.1121/1.1906346

[ref117] Livingstone M. S. (1996). Oscillatory firing and interneuronal correlations in squirrel monkey striate cortex. J. Neurophysiol. 75, 2467–2485. doi: 10.1152/jn.1996.75.6.2467, 8793757

[ref118] Löffler H. Bahmer A. (2026). The wave-coded brain: transforming spike patterns through space-time dynamics. Biol. Cybern. 120:9. doi: 10.1007/s00422-026-01034-8, 41838214

[ref119] Lohmann A. (1956). Optische Einseitenbandubertragung angewandt auf das Gabor-Mikroskop. Opt. Acta 3, 97–100.

[ref120] Longuet-Higgins H. C. (1968a). A holographic model of temporal recall. Nature 217:104. doi: 10.1038/217104a0, 5635629

[ref121] Longuet-Higgins H. C. (1968b). The non-local storage of temporal information. Proc. R. Soc. Lond. B Biol. Sci. 171, 327–334. doi: 10.1098/rspb.1968.0074 4387408

[ref122] Longuet-Higgins H. C. (1989). “A Mechanism for the Storage of Temporal Correlations,” in The Computing Neuron, eds. Durbin R. Miall C. Mitchison G. (Wokingham: Addison-Wesley), 99–104.

[ref123] Lowet E. De Weerd P. Roberts M. J. Hadjipapas A. (2022). Tuning neural synchronization: the role of variable oscillation frequencies in neural circuits. Front. Syst. Neurosci. 16:908665. doi: 10.3389/fnsys.2022.908665, 35873098 PMC9304548

[ref124] Lowet E. Roberts M. J. Bonizzi P. Karel J. De Weerd P. (2016). Quantifying neural oscillatory synchronization: a comparison between spectral coherence and phase-locking value approaches. PLoS One 11:e0146443. doi: 10.1371/journal.pone.0146443, 26745498 PMC4706353

[ref125] Luczak A. McNaughton B. L. Harris K. D. (2015). Packet-based communication in the cortex. Nat. Rev. Neurosci. 16, 745–755. doi: 10.1038/nrn4026, 26507295

[ref126] Luff C. E. Peach R. Mallas E. J. Rhodes E. Laumann F. Boyden E. S. . (2024). The neuron mixer and its impact on human brain dynamics. Cell Rep. 43:114274. doi: 10.1016/j.celrep.2024.114274, 38796852

[ref127] Lundqvist M. Miller E. K. Nordmark J. Liljefors J. Herman P. (2024). Beta: bursts of cognition. Trends Cogn. Sci. 28, 662–676. doi: 10.1016/j.tics.2024.03.010, 38658218

[ref128] Lundqvist M. Rose J. Herman P. Brincat S. L. Buschman T. J. Miller E. K. (2016). Gamma and beta bursts underlie working memory. Neuron 90, 152–164. doi: 10.1016/j.neuron.2016.02.028, 26996084 PMC5220584

[ref129] Lundqvist M. Wutz A. (2022). New methods for oscillation analyses push new theories of discrete cognition. Psychophysiology 59:e13827. doi: 10.1111/psyp.13827, 33942323 PMC11475370

[ref130] Luo H. Wang Y. Poeppel D. Simon J. Z. (2007). Concurrent encoding of frequency and amplitude modulation in human auditory cortex: encoding transition. J. Neurophysiol. 98, 3473–3485. doi: 10.1152/jn.00342.2007, 17898148

[ref131] Ma Y. (2023). Single-sideband suppressed-carrier modulation and demodulation: principles, analysis, circuits, and applications. Acad. J. Sci. Technol. 6, 147–156. doi: 10.54097/ajst.v6i3.10658

[ref132] Mackevicius E. L. Best M. D. Saal H. P. Bensmaia S. J. (2012). Millisecond precision spike timing shapes tactile perception. J. Neurosci. 32, 15309–15317. doi: 10.1523/jneurosci.2161-12.2012, 23115169 PMC3752122

[ref133] Marinković K. Dhond R. P. Dale A. M. Glessner M. Carr V. Halgren E. (2003). Spatiotemporal dynamics of modality-specific and supramodal word processing. Neuron 38, 487–497. doi: 10.1016/s0896-6273(03)00197-1, 12741994 PMC3746792

[ref134] Markram H. Gerstner W. Sjostrom P. J. (2011). A history of spike-timing-dependent plasticity. Front Synaptic Neurosci 3:4. doi: 10.3389/fnsyn.2011.00004, 22007168 PMC3187646

[ref135] Markram H. Gerstner W. Sjostrom P. J. (2012). Spike-timing-dependent plasticity: a comprehensive overview. Front Synaptic Neurosci 4:2. doi: 10.3389/fnsyn.2012.00002, 22807913 PMC3395004

[ref136] ed. Marr D. (1991). From the Retina to the Neocortex: Selected Papers of David Marr. Boston: Birkhäuser.

[ref137] Masquelier T. Hugues E. Deco G. Thorpe S. J. (2009). Oscillations, phase-of-firing coding, and spike timing-dependent plasticity: an efficient learning scheme. J. Neurosci. 29, 13484–13493. doi: 10.1523/JNEUROSCI.2207-09.2009, 19864561 PMC6665016

[ref138] McCulloch W. S. (1946). A heterarchy of values determined by the topology of nervous nets. Bull. Math. Biophys. 7, 89–93. doi: 10.1007/BF02478457 21006853

[ref139] Mitchell-Heggs R. Prado S. Gava G. P. Go M. A. Schultz S. R. (2023). Neural manifold analysis of brain circuit dynamics in health and disease. J. Comput. Neurosci. 51, 1–21. doi: 10.1007/s10827-022-00839-3, 36522604 PMC9840597

[ref140] Mohan U. R. Zhang H. Ermentrout B. Jacobs J. (2024). The direction of theta and alpha travelling waves modulates human memory processing. Nat. Hum. Behav. 8, 1124–1135. doi: 10.1038/s41562-024-01838-3, 38459263

[ref141] Mohanta S. Cleveland D. M. Afrasiabi M. Rhone A. E. Gorska U. Cooper Borkenhagen M. . (2024). Traveling waves shape neural population dynamics enabling predictions and internal model updating. bioRxiv 2024:574848. doi: 10.1101/2024.01.09.574848, 38260606 PMC10802392

[ref142] Molinaro N. Lizarazu M. (2018). Delta(but not theta)-band cortical entrainment involves speech-specific processing. Eur. J. Neurosci. 48, 2642–2650. doi: 10.1111/ejn.13811, 29283465

[ref143] Moore J. J. Ravassard P. M. Ho D. Acharya L. Kees A. L. Vuong C. . (2017). Dynamics of cortical dendritic membrane potential and spikes in freely behaving rats. Science 355:497. doi: 10.1126/science.aaj1497, 28280248

[ref144] Mountcastle V. (1967). “The Problem of Sensing and the Neural Coding of Sensory Events,” in The Neurosciences: A Study Program, eds. Quarton G. C. Melnechuk T. Schmitt F. O. (New York: Rockefeller University Press).

[ref145] Muller L. Chavane F. Reynolds J. Sejnowski T. J. (2018). Cortical travelling waves: mechanisms and computational principles. Nat. Rev. Neurosci. 19, 255–268. doi: 10.1038/nrn.2018.20, 29563572 PMC5933075

[ref146] Muller L. Piantoni G. Koller D. Cash S. S. Halgren E. Sejnowski T. J. (2016). Rotating waves during human sleep spindles organize global patterns of activity that repeat precisely through the night. eLife 5:17267. doi: 10.7554/eLife.17267, 27855061 PMC5114016

[ref147] Myers J. C. Smith E. H. Leszczynski M. O'Sullivan J. Yates M. J. McKhann G. . (2022). The spatial reach of neuronal coherence and spike-field coupling across the human neocortex. J. Neurosci. 42, 6285–6294. doi: 10.1523/jneurosci.0050-22.2022, 35790403 PMC9374135

[ref148] Myrov V. Siebenhuhner F. Juvonen J. J. Arnulfo G. Palva S. Palva J. M. (2024). Rhythmicity of neuronal oscillations delineates their cortical and spectral architecture. Commun. Biol. 7:405. doi: 10.1038/s42003-024-06083-y, 38570628 PMC10991572

[ref149] Nadel L. Maurer A. P. (2020). Recalling Lashley and reconsolidating Hebb. Hippocampus 30, 776–793. doi: 10.1002/hipo.23027, 30216593 PMC6417981

[ref150] Nahin P. J. (2024). The Mathematical Radio: Inside the Magic of AM, FM, and Single-Sideband. Princeton, NJ: Princeton University Press.

[ref151] Nickel M. Rosasco L. Poggio T. (2016). *Holographic Embeddings of Knowledge Graphs*. AAAI-16: Proceedings of the Thirtieth AAAI Conference on Artificial Intelligence, pp. 1955–1961.

[ref152] Orepic P. Truccolo W. Halgren E. Cash S. S. Giraud A. L. Proix T. (2024). Neural manifolds carry reactivation of phonetic representations during semantic processing. bioRxiv 2024:638. doi: 10.1101/2023.10.30.564638

[ref153] Oswald A. A. (1956). Early history of single-sideband transmission. Proc. IRE 44, 1676–1679. doi: 10.1109/JRPROC.1956.275033

[ref154] Padamsey Z. Rochefort N. L. (2023). Paying the brain's energy bill. Curr. Opin. Neurobiol. 78:102668. doi: 10.1016/j.conb.2022.102668, 36571958

[ref155] Palva J. M. Palva S. Kaila K. (2005). Phase synchrony among neuronal oscillations in the human cortex. J. Neurosci. 25, 3962–3972. doi: 10.1523/JNEUROSCI.4250-04.2005, 15829648 PMC6724920

[ref156] Panzeri S. Engel N. M. Celotto M. (2026). Contribution of spike timing to the neural code: from fast to slow timescales. Biol. Cybern. 120:12. doi: 10.1007/s00422-026-01042-8, 41979854 PMC13079490

[ref157] Penttonen M. Buzsáki G. (2003). Natural logarithmic relationship between brain oscillators. Thalamus Relat. Syst. 2, 145–152. doi: 10.1017/S1472928803000074, 41292463

[ref158] Perich M. G. Narain D. Gallego J. A. (2025). A neural manifold view of the brain. Nat. Neurosci. 28, 1582–1597. doi: 10.1038/s41593-025-02031-z, 40721675

[ref159] Perkell D. H. Bullock T. H. (1968). Neural coding. Neurosci. Res. Program Bull. 6, 221–348.

[ref160] Phillips D. P. (1993). Neural representation of stimulus times in the primary auditory cortex. Ann. N. Y. Acad. Sci. 682, 104–118. doi: 10.1111/j.1749-6632.1993.tb22963.x, 8323107

[ref161] Pietsch P. (1981). Shufflebrain. Boston: Houghton Mifflin.

[ref162] Plate T. A. (2003). Holographic Reduced Representation: Distributed Representation for Cognitive Structures. Stanford, Calif: CSLI Publications.

[ref163] Pribram K. H. (1971). Languages of the Brain: Experimental Paradoxes and Principles in Neurophysiology. New York: Prentice-Hall.

[ref164] Pribram K. H. (2013). The Form within: My Point of View. Westport, CT: Prospecta Press.

[ref165] Rao R. P. Sejnowski T. J. (2001). Spike-timing-dependent Hebbian plasticity as temporal difference learning. Neural Comput. 13, 2221–2237. doi: 10.1162/089976601750541787, 11570997

[ref166] Reichardt W. (1957). Autokorrelationsauswertung als Funktionsprincip des Zentalnervensystem. Z. Naturforsch. 12b:448.

[ref167] Reichardt W. (1961). “Autocorrelation, a Principle for the Evaluation of Sensory Information by the Central Nervous System,” in Sensory Communication, ed. Rosenblith W. A. (New York: MIT Press/John Wiley), 303–317.

[ref168] Rossiter H. E. Davis E. M. Clark E. V. Boudrias M. H. Ward N. S. (2014). Beta oscillations reflect changes in motor cortex inhibition in healthy ageing. NeuroImage 91, 360–365. doi: 10.1016/j.neuroimage.2014.01.012, 24440529 PMC3988925

[ref169] Roy A. E. (1960). On a method of storing information. Bull. Math. Biophys. 22, 139–168. doi: 10.1007/bf02478003

[ref170] Roy A. E. (1962). On a method of storing information: II. Further study of model properties. Bull. Math. Biophys. 24, 39–50.

[ref171] Safavi S. Panagiotaropoulos T. I. Kapoor V. Ramirez-Villegas J. F. Logothetis N. K. Besserve M. (2023). Uncovering the organization of neural circuits with generalized phase locking analysis. PLoS Comput. Biol. 19:e1010983. doi: 10.1371/journal.pcbi.1010983, 37011110 PMC10109521

[ref172] Salimpour Y. Anderson W. S. (2019). Cross-frequency coupling based neuromodulation for treating neurological disorders. Front. Neurosci. 13:125. doi: 10.3389/fnins.2019.00125, 30846925 PMC6393401

[ref173] Sauseng P. Klimesch W. (2008). What does phase information of oscillatory brain activity tell us about cognitive processes? Neurosci. Biobehav. Rev. 32, 1001–1013. doi: 10.1016/j.neubiorev.2008.03.014, 18499256

[ref174] Schneider M. Broggini A. C. Dann B. Tzanou A. Uran C. Sheshadri S. . (2021). A mechanism for inter-areal coherence through communication based on connectivity and oscillatory power. Neuron 109, 4050–4067.e12. doi: 10.1016/j.neuron.2021.09.037, 34637706 PMC8691951

[ref175] Sejnowski T. J. (2026). Dynamical Mechanisms for Coordinating long-term Working memory based on the Precision of Spike-Timing in cortical Neurons. ArXiv 2026:15891. doi: 10.48550/arXiv.2512.15891 PMC13371168 42126616

[ref176] Shervani-Tabar N. Brincat S. L. Lundqvist M. Miller E. K. (2026). Emergent traveling waves in neural circuits. bioRxiv 2026:698281. doi: 10.64898/2026.01.08.698281

[ref177] Shoidin S. A. Pazoev A. L. (2021). Remote formation of holographic record. Optoelectron. Instrument. Proc. 57, 80–88. doi: 10.3103/S8756699021010118

[ref178] Shoydin S. A. Pazoev A. L. (2021). Transmission of 3d holographic information via conventional communication channels and the possibility of multiplexing in the implementation of 3d hyperspectral images. Photonics 8:448. doi: 10.3390/photonics8100448

[ref179] Siegel M. Warden M. R. Miller E. K. (2009). Phase-dependent neuronal coding of objects in short-term memory. Proc. Natl. Acad. Sci. U. S. A. 106, 21341–21346. doi: 10.1073/pnas.0908193106, 19926847 PMC2779828

[ref180] Siems M. Cao Y. Donner T. H. Tsetsos K. Engel A. K. (2025). Inter-areal coupling for cognition through coincident oscillatory transients. bioRxiv 2025:689181. doi: 10.1101/2025.11.21.689181

[ref181] Singer W. (1999). Time as coding space? Curr. Opin. Neurobiol. 9, 189–194. doi: 10.1016/S0959-4388(99)80026-9, 10322191

[ref182] Singer W. Effenberger F. (2025). Oscillations in natural neuronal networks; an epiphenomenon or a fundamental computational mechanism? Hum. Arenas 8, 846–868. doi: 10.1007/s42087-025-00478-x

[ref183] Song S. Miller K. D. Abbott L. F. (2000). Competitive Hebbian learning through spike-timing-dependent synaptic plasticity. Nat. Neurosci. 3, 919–926. doi: 10.1038/78829, 10966623

[ref184] Thorpe S. J. Guyonneau R. Guilbaud N. Allegraud J.-M. VanRullen R. (2004). SpikeNet: real time visual processing with one spike per neuron. Neurocomputing 58-60, 857–864. doi: 10.1016/j.neucom.2004.01.138

[ref185] Tort A. B. Komorowski R. Eichenbaum H. Kopell N. (2010). Measuring phase-amplitude coupling between neuronal oscillations of different frequencies. J. Neurophysiol. 104, 1195–1210. doi: 10.1152/jn.00106.2010, 20463205 PMC2941206

[ref186] van Bree S. Levenstein D. Krause M. R. Voytek B. Gao R. (2025). Processes and measurements: a framework for understanding neural oscillations in field potentials. Trends Cogn. Sci. 29, 448–466. doi: 10.1016/j.tics.2024.12.003, 39753446

[ref187] Van Heerden P. J. (1970). Models for the brain. Nature 227, 410–411. 10.1038/227410b0 5428449

[ref188] VanRullen R. Thorpe S. J. (2001). The time course of visual processing: from early perception to decision-making. J. Cogn. Neurosci. 13, 454–461. doi: 10.1162/08989290152001880, 11388919

[ref189] Varela F. Lachaux J. P. Rodriguez E. Martinerie J. (2001). The brainweb: phase synchronization and large-scale integration. Nat. Rev. Neurosci. 2, 229–239. doi: 10.1038/35067550, 11283746

[ref190] Von der Malsburg C. (1999). The what and why of binding: the modeler's perspective. Neuron 24, 95–104. 10677030 10.1016/s0896-6273(00)80825-9

[ref191] Wallace E. Benayoun M. van Drongelen W. Cowan J. D. (2011). Emergent oscillations in networks of stochastic spiking neurons. PLoS One 6:e14804. doi: 10.1371/journal.pone.0014804, 21573105 PMC3089610

[ref192] Weiss S. Mueller H. M. (2012). "too many betas do not spoil the broth": the role of beta brain oscillations in language processing. Front. Psychol. 3:201. doi: 10.3389/fpsyg.2012.00201, 22737138 PMC3382410

[ref193] Westlake P. R. (1968). A Detailed Investigation of the Possibilities of Neural Holographic Processes. Ph.D., University of California, Los Angeles. Ann Arbor, Michigan: University Microfilms.

[ref194] Westlake P. R. (1970). The possibilities of neural holographic processes within the brain. Kybernetik 7, 129–153. doi: 10.1007/BF00571694, 5512392

[ref195] Willshaw D. J. Buneman O. P. Longuet-Higgins H. C. (1969). A non-holographic model of associative memory. Nature 222, 960–962. 5789326 10.1038/222960a0

[ref196] Womelsdorf T. Fries P. (2006). Neuronal coherence during selective attentional processing and sensory-motor integration. J. Physiol. Paris 100, 182–193. doi: 10.1016/j.jphysparis.2007.01.005, 17317118

[ref197] Womelsdorf T. Schoffelen J. M. Oostenveld R. Singer W. Desimone R. Engel A. K. . (2007). Modulation of neuronal interactions through neuronal synchronization. Science 316, 1609–1612. doi: 10.1126/science.1139597, 17569862

[ref198] Xie W. Wittig J. H. Chapeton J. I. El-Kalliny M. Jackson S. N. Inati S. K. . (2024). Neuronal sequences in population bursts encode information in human cortex. Nature 635, 935–942. doi: 10.1038/s41586-024-08075-8, 39415012 PMC12279075

[ref199] Xie J. Yan T. Zhang J. Ma Z. Zhou H. (2022). Modulation of neuronal activity and saccades at theta rhythm during visual search in non-human primates. Neurosci. Bull. 38, 1183–1198. doi: 10.1007/s12264-022-00884-z, 35608752 PMC9554076

[ref200] Yeh C. H. Zhang C. Shi W. Lo M. T. Tinkhauser G. Oswal A. (2023). Cross-frequency coupling and intelligent neuromodulation. Cyborg Bionic Syst 4:0034. doi: 10.34133/cbsystems.0034, 37266026 PMC10231647

[ref201] Yiling Y. Shapcott K. Peter A. Klon-Lipok J. Xuhui H. Lazar A. . (2023). Robust encoding of natural stimuli by neuronal response sequences in monkey visual cortex. Nat. Commun. 14:3021. doi: 10.1038/s41467-023-38587-2, 37231014 PMC10212951

[ref202] Zhang M. Chowdhury S. Saggar M. (2023). Temporal mapper: transition networks in simulated and real neural dynamics. Netw. Neurosci. 7, 431–460. doi: 10.1162/netn_a_00301, 37397880 PMC10312258

[ref203] Zhang H. Watrous A. J. Patel A. Jacobs J. (2018). Theta and alpha oscillations are traveling waves in the human neocortex. Neuron 98, 1269–1281.e4. doi: 10.1016/j.neuron.2018.05.019, 29887341 PMC6534129

